# Supplementary material for: Modeling Enzyme Processivity Reveals that RNA-Seq Libraries Are Biased in Characteristic and Correctable Ways
Source: Cell Syst. 2016 Nov 23;3(5):467–479.e12. doi: 10.1016/j.cels.2016.10.012 (PMC5167349; doi:10.1016/j.cels.2016.10.012)
Supplement: Document S1. Figures S1–S8 and Tables S1 and S2 [file mmc1.pdf]

Cell Systems, Volume *3*

Supplemental Information

Modeling Enzyme Processivity Reveals that RNA-Seq Libraries Are Biased in Characteristic and Correctable Ways

Nathan Archer, Mark D. Walsh, Vahid Shahrezaei, and Daniel Hebenstreit

SUPPLEMENTAL FIGURES

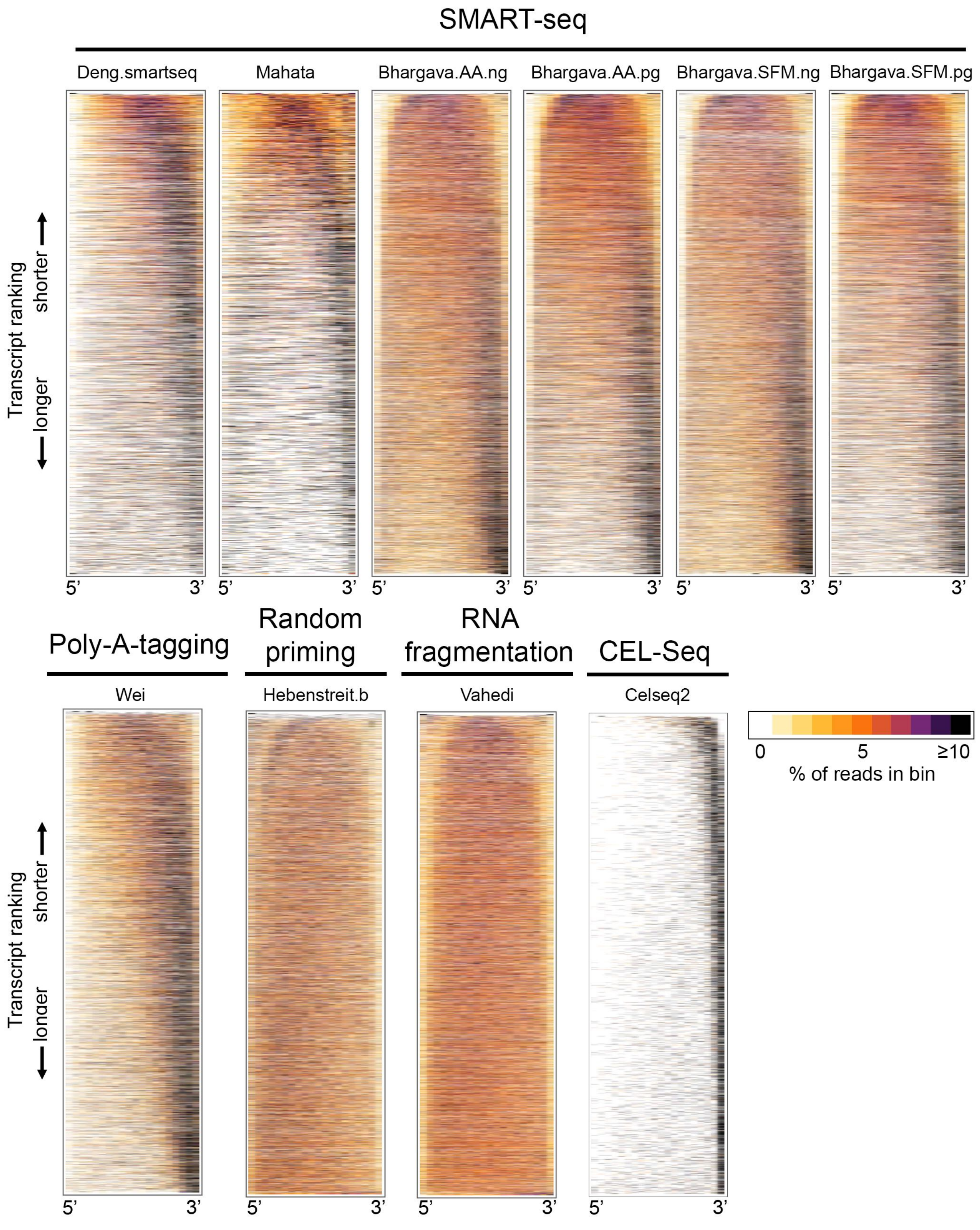

Figure S1. **Related to Figure 1. Related to Table 1**. RNA-seq coverage along transcripts for different datasets as in Figure 1C. Details of the datasets shown are listed in Table 1.

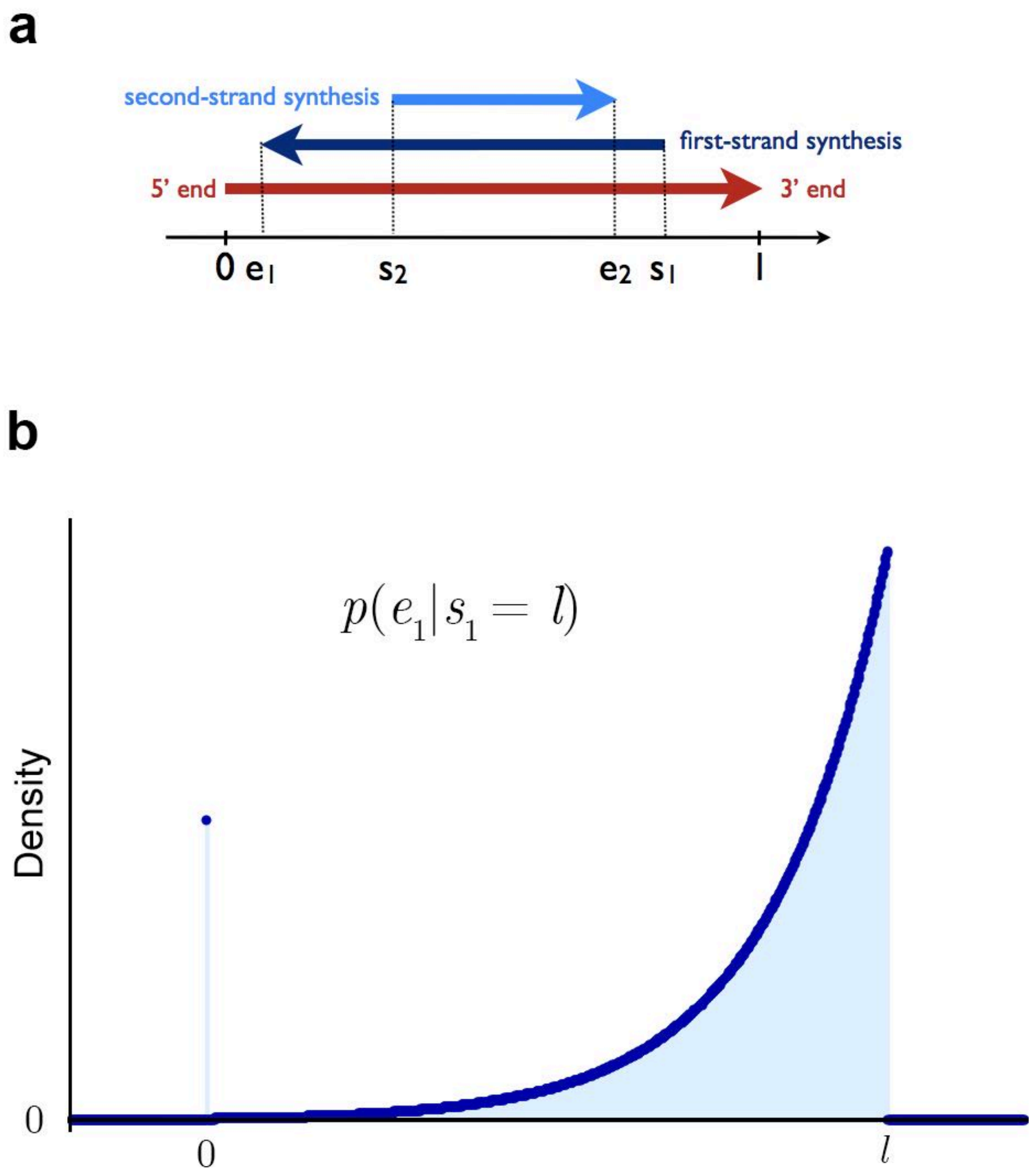

Figure S2. Related to Figure 1. Related to Derivation of models (method details). (**a**) Illustration of mRNA and first- and second-strand synthesis. (**b**) Graph of a typical stopping density for first-strand synthesis. If synthesis is based on poly-A tail priming, stopping positions decrease exponentially from the 3' end until the 5' end is reached, giving rise to a spike at the 5' end (position 0). The density equals zero outside the transcript length. Analogous the graph for $p(e_2 \mid s_2)$, mirrored horizontally and with different starting positions.

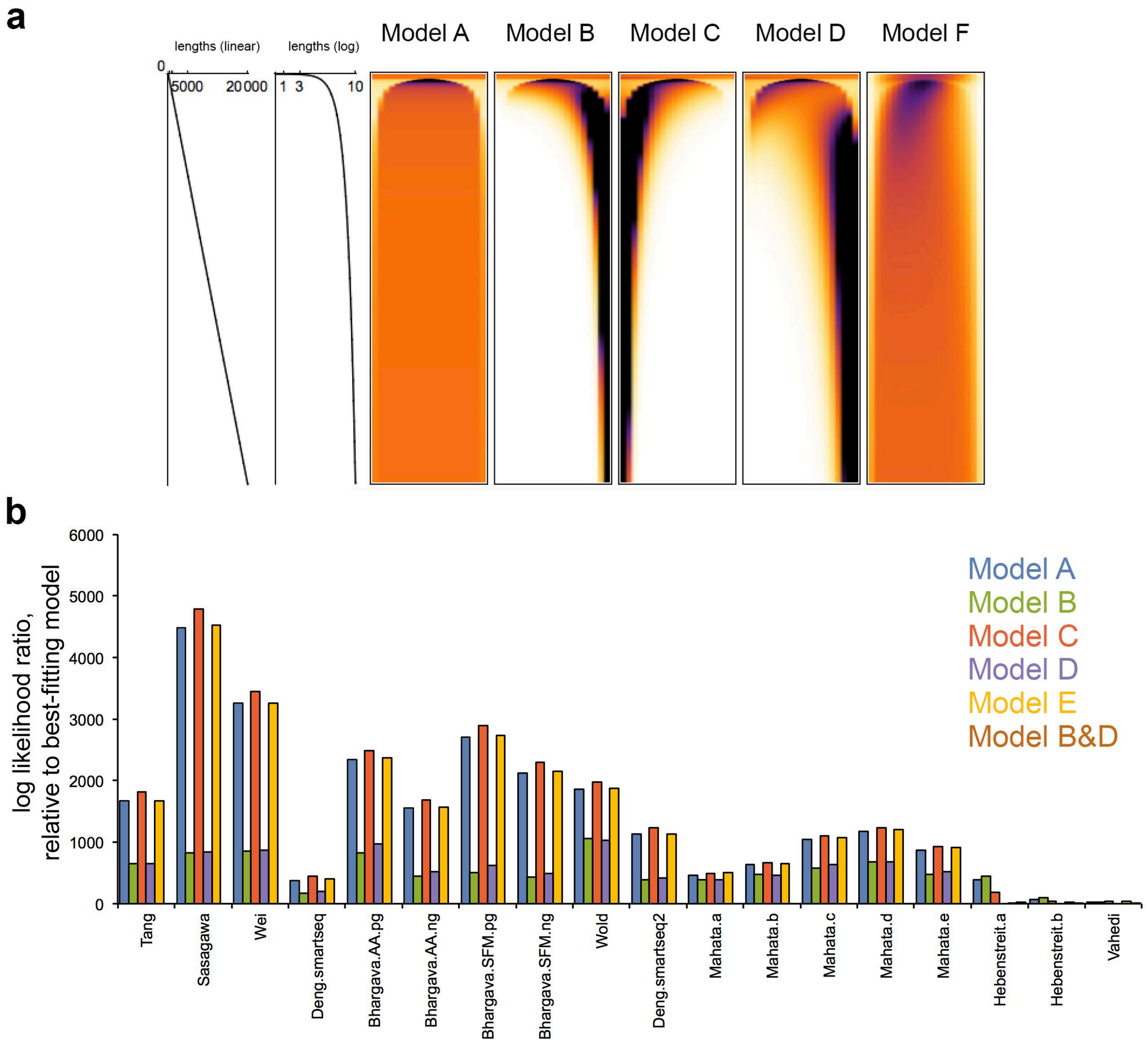

Figure S3. Related to Figure 2. (**a**) Coverage heatmaps for the different models A to E as in Figure 1C, using a transcript length distribution that linearly increases from 1 bp to 20 kb (bottom row). (**b**) Log likelihood ratios with respect to the best-fitting models for all models and relevant datasets used in this study. Best-fitting model in most cases is model B&D. The lower the value, the better the fit compared to the best model.

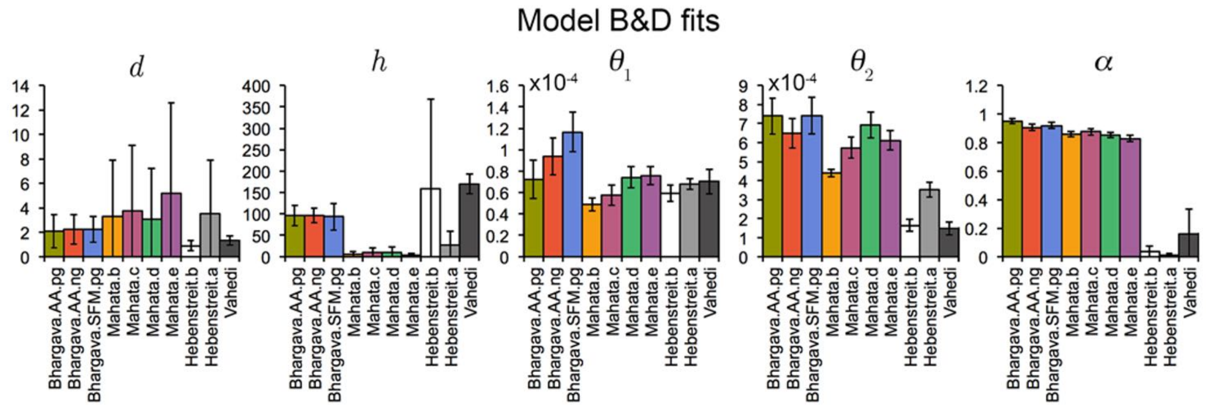

Figure S4. Related to Figure 2. MCMC parameter estimates for model B&D for datasets not shown in Figure 2C.The bar heights correspond to the medians, the error bars correspond to the median absolute deviations.

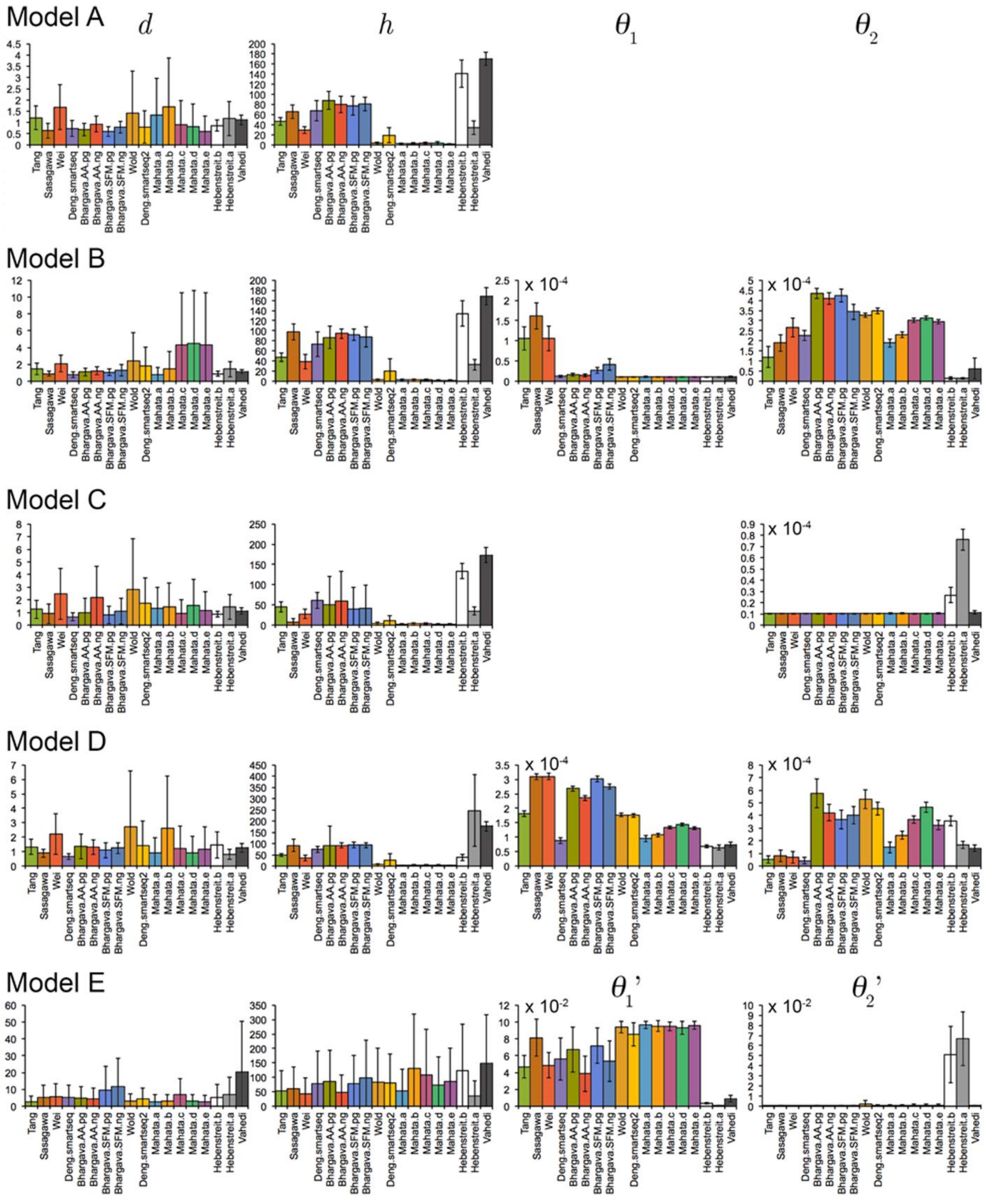

Figure S5. Related to Figure 2. MCMC parameter estimates for all relevant datasets and models except model B&D (Figure 2C;Figure S4). The bar heights correspond to the medians, the error bars correspond to the median absolute deviations.

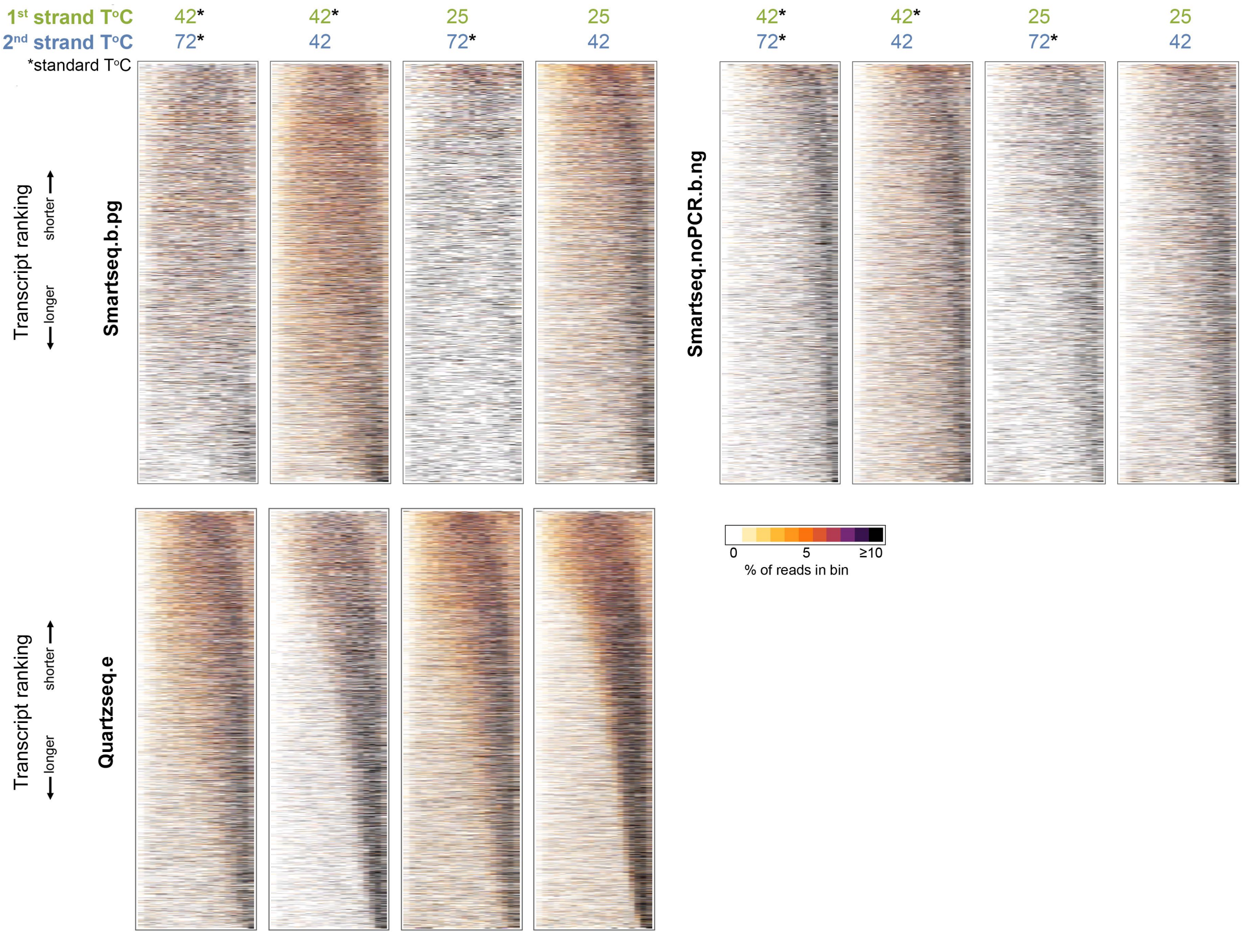

Figure S6. Related to Figure 3. Coverage heatmaps as in Figure 1C for a selection of RNA-seq samples prepared in this study as indicated. Transcripts are ordered by length. Four different combinations of incubation temperatures for first- and second-strand synthesis were used as indicated. 'Standard' temperatures are 42°C and 72°C for first- and second-strand, respectively, which were lowered to 25°C and 42°C, respectively, in the designated samples.

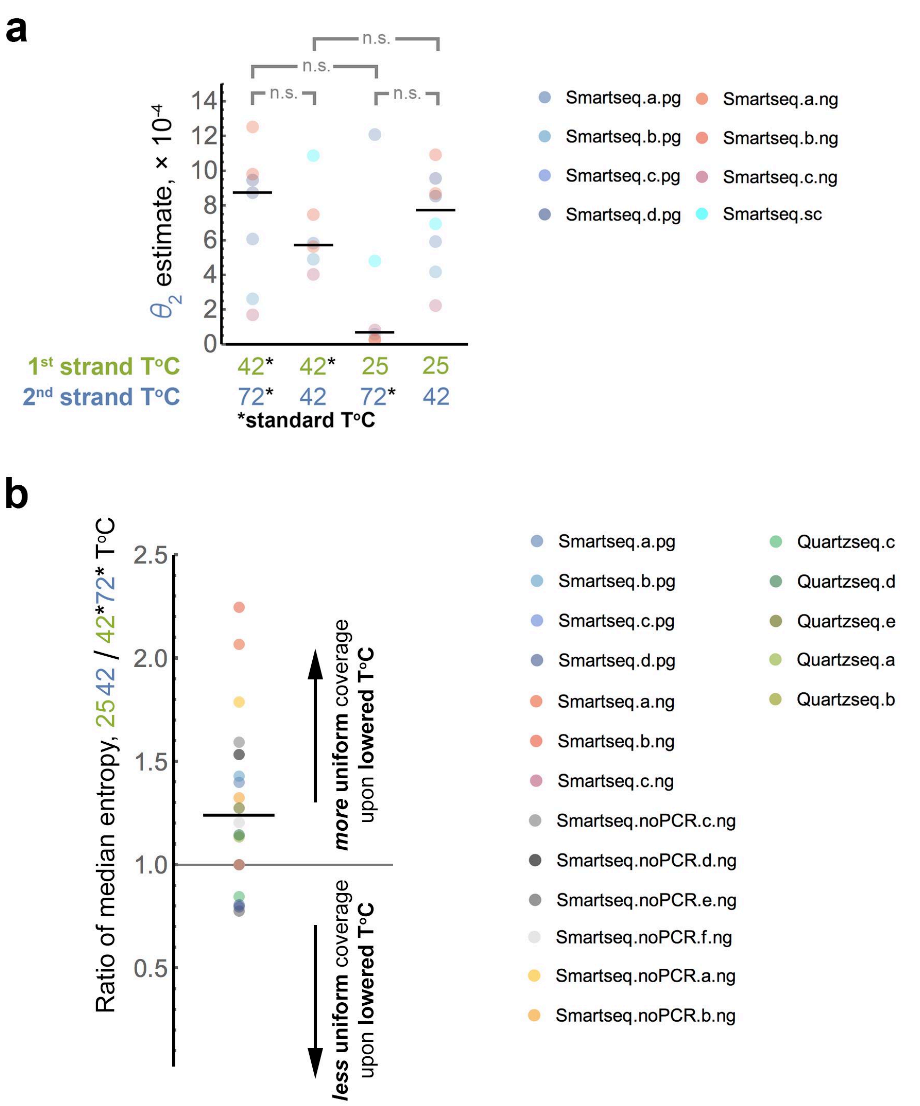

Figure S7. Related to Figure 3; Figure 4. (**a**) MCMC parameter estimates for Θ_2 for diverse SMART-seq samples (Table S1, color code on right of figure) prepared with altered reaction temperatures during first- and second-strand syntheses. 'Standard' temperatures were 42°C and 72°C for first- and second-strand, respectively, which were lowered to 25°C and 42°C, respectively, in the designated samples (black horizontal lines indicate the median; n.s., one-sided Wilcoxon signed-rank tests). (**b**) Ratios of the average coverage uniformities between lowered incubation temperatures for first- and second-strand (25°C and 42°C, respectively) and 'standard' temperatures (42°C and 72°C, respectively). Sequencing reads along transcripts were binned in the same way as for the heatmaps (e.g. Figure 1c), and statistical entropy (as a measure of uniformity) was calculated for each transcript. The statistical entropy becomes maximal for uniform distributions. The medians of the resulting distributions were determined and are shown as ratios for the RNA-seq samples as indicated.

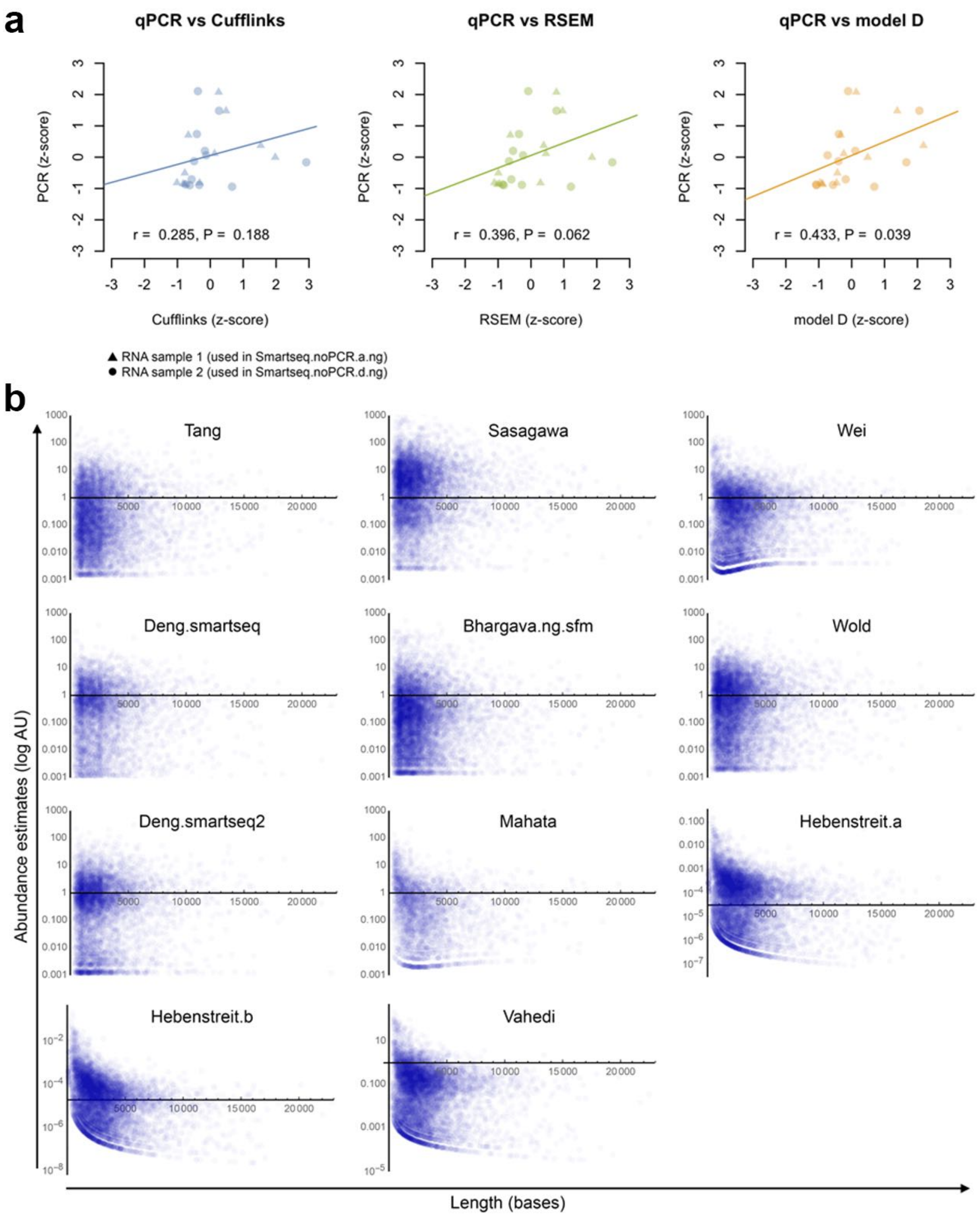

Figure S8. Related to Figure 4. (**a**) Comparison of RNA-seq abundance estimates with qPCR-based quantification. RNA-seq samples deriving from two different RNA samples (Table S1) were quantified by RSEM, Cufflinks, or our Model D as indicated for twelve different genes that cover a wide range of mRNA lengths (Table S2). In parallel, the corresponding two RNA samples (dots and triangle symbols, respectively) were subjected to qPCR for the same twelve genes. Each dot corresponds to one gene. Fitted trend lines and

Pearson's product moment correlation coefficient (*r*) and its *P*-value are shown. Standardized measures (z-scores) are used to make the approaches comparable. (**b**) Abundance estimates vs. transcript lengths based on fitting our models. Vahedi (RNA-fragmentation) was fit with model A, Hebenstreit.a/b (random priming) with model E, all others (poly-A-tagging and SMART-seq) with model B&D.

SUPPLEMENTAL TABLES

Sample name	1st strand T°C	2nd strand T°C	Protocol	Tissue	Starting Material	used in qPCR & RNA-seq analysis	Used in θ_1 pertur-bation analysis	Used in θ_2 pertur-bation analysis	Used in sensitivity analysis (spike-in probes)	Used in length ratio & local bias analysis	Read numbers ($\times 10^4$)
Quartzseq.a.r25ss42	25	42	Quartz-seq	Liver	100 ng Poly(A)+		x	x	x	x	903
Quartzseq.a.r25ss72	25	72	Quartz-seq	Liver	100 ng Poly(A)+		x	x			827
Quartzseq.a.r42ss42	42	42	Quartz-seq	Liver	100 ng Poly(A)+		x	x			501
Quartzseq.a.r42ss72	42	72	Quartz-seq	Liver	100 ng Poly(A)+		x	x	x	x	801
Quartzseq.b.r25ss42	25	42	Quartz-seq	Lymphocyte	100 ng Poly(A)+		x	x	x	x	1051
Quartzseq.b.r25ss72	25	72	Quartz-seq	Lymphocyte	100 ng Poly(A)+		x	x			1452
Quartzseq.b.r42ss42	42	42	Quartz-seq	Lymphocyte	100 ng Poly(A)+		x	x			0.44
Quartzseq.b.r42ss72	42	72	Quartz-seq	Lymphocyte	100 ng Poly(A)+		x	x	x	x	1554
Quartzseq.c.r25ss42	25	42	Quartz-seq	Lymphocyte	100 ng Poly(A)+		x	x	x	x	1691
Quartzseq.c.r25ss72	25	72	Quartz-seq	Lymphocyte	100 ng Poly(A)+		x	x			1447
Quartzseq.c.r42ss42	42	42	Quartz-seq	Lymphocyte	100 ng Poly(A)+		x	x			1922
Quartzseq.c.r42ss72	42	72	Quartz-seq	Lymphocyte	100 ng Poly(A)+		x	x	x	x	813
Quartzseq.d.r25ss42	25	42	Quartz-seq	Lymphocyte	100 ng Poly(A)+		x	x	x	x	2407
Quartzseq.d.r25ss72	25	72	Quartz-seq	Lymphocyte	100 ng Poly(A)+		x	x			2519
Quartzseq.d.r42ss42	42	42	Quartz-seq	Lymphocyte	100 ng Poly(A)+		x	x			649
Quartzseq.d.r42ss72	42	72	Quartz-seq	Lymphocyte	100 ng Poly(A)+		x	x	x	x	1753
Quartzseq.e.r25ss42	25	42	Quartz-seq	Lymphocyte	100 ng Poly(A)+		x	x	x	x	1354
Quartzseq.e.r25ss72	25	72	Quartz-seq	Lymphocyte	100 ng Poly(A)+		x	x			1255
Quartzseq.e.r42ss42	42	42	Quartz-seq	Lymphocyte	100 ng Poly(A)+		x	x			743
Quartzseq.e.r42ss72	42	72	Quartz-seq	Lymphocyte	100 ng Poly(A)+		x	x	x	x	1196
Smartseq.a.ng.r25ss42	25	42	Smart-seq	Lymphocyte	100 ng Poly(A)+		x		x	x	894
Smartseq.a.ng.r25ss72	25	72	Smart-seq	Lymphocyte	100 ng Poly(A)+		x				729
Smartseq.a.ng.r42ss42	42	42	Smart-seq	Lymphocyte	100 ng Poly(A)+		x				264
Smartseq.a.ng.r42ss72	42	72	Smart-seq	Lymphocyte	100 ng Poly(A)+		x		x	x	114
Smartseq.a.pg.r25ss42	25	42	Smart-seq 2	Liver	10 pg Total RNA		x		x	x	2297
Smartseq.a.pg.r25ss72	25	72	Smart-seq 2	Liver	10 pg Total RNA		x				928
Smartseq.a.pg.r42ss42	42	42	Smart-seq 2	Liver	10 pg Total RNA		x				3956
Smartseq.a.pg.r42ss72	42	72	Smart-seq 2	Liver	10 pg Total RNA		x		x	x	1481
Smartseq.b.ng.r25ss42	25	42	Smart-seq	Lymphocyte	100 ng Poly(A)+		x		x	x	1487
Smartseq.b.ng.r25ss72	25	72	Smart-seq	Lymphocyte	100 ng Poly(A)+		x				1190
Smartseq.b.ng.r42ss42	42	42	Smart-seq	Lymphocyte	100 ng Poly(A)+		x				1172
Smartseq.b.ng.r42ss72	42	72	Smart-seq	Lymphocyte	100 ng Poly(A)+		x		x	x	622
Smartseq.b.pg.r25ss42	25	42	Smart-seq 2	Liver	10 pg Total RNA		x		x	x	977
Smartseq.b.pg.r25ss72	25	72	Smart-seq 2	Liver	10 pg Total RNA		x				380
Smartseq.b.pg.r42ss42	42	42	Smart-seq 2	Liver	10 pg Total RNA		x				893
Smartseq.b.pg.r42ss72	42	72	Smart-seq 2	Liver	10 pg Total RNA		x		x	x	312
Smartseq.c.ng.r25ss42	25	42	Smart-seq	Lymphocyte	100 ng Poly(A)+		x		x	x	37
Smartseq.c.ng.r25ss72	25	72	Smart-seq	Lymphocyte	100 ng Poly(A)+		x				1013
Smartseq.c.ng.r42ss42	42	42	Smart-seq	Lymphocyte	100 ng Poly(A)+		x				639
Smartseq.c.ng.r42ss72	42	72	Smart-seq	Lymphocyte	100 ng Poly(A)+		x		x	x	9
Smartseq.c.pg.r25ss42	25	42	Smart-seq 2	Liver	10 pg Total RNA		x		x	x	1159
Smartseq.c.pg.r42ss72	42	72	Smart-seq 2	Liver	10 pg Total RNA		x		x	x	871
Smartseq.d.pg.r25ss42	25	42	Smart-seq 2	Liver	10 pg Total RNA		x		x	x	2730
Smartseq.d.pg.r42ss72	42	72	Smart-seq 2	Liver	10 pg Total RNA		x		x	x	1200
Smartseq.noPCR.a.ng.r25ss42	25	42	Smart-seq, no PCR	Lymphocyte	100 ng Poly(A)+	x	x	x	x	x	1530
Smartseq.noPCR.a.ng.r25ss72	25	72	Smart-seq, no PCR	Lymphocyte	100 ng Poly(A)+	x	x	x			445

Smartseq.noPCR.a.ng.r42ss42	42	42	Smart-seq, no PCR	Lymphocyte	100 ng Poly(A)+	x	x	x			836
Smartseq.noPCR.a.ng.r42ss72	42	72	Smart-seq, no PCR	Lymphocyte	100 ng Poly(A)+	x	x	x	x	x	452
Smartseq.noPCR.b.ng.r25ss42	25	42	Smart-seq, no PCR	Lymphocyte	100 ng Poly(A)+		x	x	x	x	1166
Smartseq.noPCR.b.ng.r25ss72	25	72	Smart-seq, no PCR	Lymphocyte	100 ng Poly(A)+		x	x			893
Smartseq.noPCR.b.ng.r42ss42	42	42	Smart-seq, no PCR	Lymphocyte	100 ng Poly(A)+		x	x			1767
Smartseq.noPCR.b.ng.r42ss72	42	72	Smart-seq, no PCR	Lymphocyte	100 ng Poly(A)+		x	x	x	x	1836
Smartseq.noPCR.c.ng.r25ss42	25	42	Smart-seq, no PCR	Lymphocyte	1 µg Total RNA		x	x		x	13523
Smartseq.noPCR.c.ng.r25ss72	25	72	Smart-seq, no PCR	Lymphocyte	1 µg Total RNA		x	x			3996
Smartseq.noPCR.c.ng.r42ss42	42	42	Smart-seq, no PCR	Lymphocyte	1 µg Total RNA		x	x			4689
Smartseq.noPCR.c.ng.r42ss72	42	72	Smart-seq, no PCR	Lymphocyte	1 µg Total RNA		x	x		x	10196
Smartseq.noPCR.d.ng.r25ss42	25	42	Smart-seq, no PCR	Lymphocyte	100 ng Poly(A)+	x	x	x		x	5744
Smartseq.noPCR.d.ng.r25ss72	25	72	Smart-seq, no PCR	Lymphocyte	100 ng Poly(A)+	x	x	x			2880
Smartseq.noPCR.d.ng.r42ss42	42	42	Smart-seq, no PCR	Lymphocyte	100 ng Poly(A)+	x	x	x			5635
Smartseq.noPCR.d.ng.r42ss72	42	72	Smart-seq, no PCR	Lymphocyte	100 ng Poly(A)+	x	x	x		x	2420
Smartseq.noPCR.e.ng.r25ss42	25	42	Smart-seq, no PCR	Lymphocyte	100 ng Poly(A)+		x	x		x	3598
Smartseq.noPCR.e.ng.r25ss72	25	72	Smart-seq, no PCR	Lymphocyte	100 ng Poly(A)+		x	x			3558
Smartseq.noPCR.e.ng.r42ss42	42	42	Smart-seq, no PCR	Lymphocyte	100 ng Poly(A)+		x	x			4226
Smartseq.noPCR.e.ng.r42ss72	42	72	Smart-seq, no PCR	Lymphocyte	100 ng Poly(A)+		x	x		x	2753
Smartseq.noPCR.f.ng.r25ss42	25	42	Smart-seq, no PCR	Lymphocyte	100 ng Poly(A)+		x	x		x	3424
Smartseq.noPCR.f.ng.r25ss72	25	72	Smart-seq, no PCR	Lymphocyte	100 ng Poly(A)+		x	x			4847
Smartseq.noPCR.f.ng.r42ss42	42	42	Smart-seq, no PCR	Lymphocyte	100 ng Poly(A)+		x	x			3639
Smartseq.noPCR.f.ng.r42ss72	42	72	Smart-seq, no PCR	Lymphocyte	100 ng Poly(A)+		x	x		x	7234
Smartseq.sc.r25ss42	25	42	Smart-seq 2	CD5+ lymphocyte	Single cell		x			x	224
Smartseq.sc.r25ss72	25	72	Smart-seq 2	CD5+ lymphocyte	Single cell		x				236
Smartseq.sc.r42ss42	42	42	Smart-seq 2	CD5+ lymphocyte	Single cell		x				179
Smartseq.sc.r42ss72	42	72	Smart-seq 2	CD5+ lymphocyte	Single cell		x			x	0.62

Table S1. Related to Figure 3. RNA-seq samples prepared in this study.

ID	**mRNA length** (bases)	**fwd primer**	**rev primer**	**amplicon length** (bases)	**distance to 3' end of mRNA** (bases)
NM_001100181.1	1913	cctcctcatcccctctcttc	gagttttggagcccatggtac	178	178
NM_001100181.1	1913	cctcctcatcccctctcttc	agttttggagcccatggtaca	177	178
NM_026389.3	1951	ggagcttctttcaggccaac	gcaagaatgaaaggacctctaac	108	131
NM_026389.3	1951	ggagcttctttcaggccaac	gtgaaggaatcctaagacctggg	72	131
NM_025989.3	1978	ttcgattgccctggaaatgc	gcaagggttgatgtattcaag	143	146
NM_025989.3	1978	ttcgattgccctggaaatgc	tgggatttgccctttcccat	83	146
NM_027949.1	1984	gcccaggacagtgagataca	ggagagatgacctttatttgtc	226	226
NM_027949.1	1984	gcccaggacagtgagataca	aggaagcacatgagagccac	178	226
NM_001081061.1	9921	cccaggcaagacatagatgc	ctacagaacattactgctttctttag	162	199
NM_001081061.1	9921	cccaggcaagacatagatgc	acatggtcacttgcatttgaataa	72	199
NM_013889.2	9977	tggattgtgttgagtagttggt	aaatcaaccatgaaaaccacc	133	151
NM_013889.2	9977	tggattgtgttgagtagttggt	acagtgggcttaccaaggat	90	151
NM_001160400.1	10040	gcttgggactcttgctttcc	gtcttctccacactttattctttg	195	197
NM_001160400.1	10040	gcttgggactcttgctttcc	tgacccatgctgacatgcac	143	197
NM_001081203.1	10070	cagggttcatttgcccacaa	caaagtgcatttgtttaattttatttaagaactttac	156	156
NM_054053.4	19327	gcattgcttagtgtttgtgca	ggattccaatacttttattgatg	171	171

NM_054053.4	19327	gcattgcttagtgtttgtgca	accatgttagccaagttcgga	134	171
NM_054053.4	19327	gcattgcttagtgtttgtgca	gattattttcattaaaccatgttagcc	149	171
NM_001033276.3	19825	ccctgtccttggcatgtttt	gtttaaaaacaaactttgaagaagcaaaatcc	114	142
NM_001033276.3	19825	ccctgtccttggcatgtttt	ccaaaacttgccctttgcct	83	142
NM_001005510.2	21718	ggtctggttgagctgtttgg	gagaaacatctgagttgacatatc	81	112
NM_010889.1	22489	accaaaccagtccttcccaa	gttgcccatgaacagtttatttc	210	210
NM_010889.1	22489	accaaaccagtccttcccaa	aaacaagggaaagggcttct	95	210

Table S2. Related to Figure 4; Related to Key Resources Table. Genes and corresponding primers used in qPCR.
