# Supplementary material for: Modeling Enzyme Processivity Reveals that RNA-Seq Libraries Are Biased in Characteristic and Correctable Ways
Source: Cell Syst. 2016 Nov 23;3(5):467–479.e12. doi: 10.1016/j.cels.2016.10.012 (PMC5167349; doi:10.1016/j.cels.2016.10.012)
Supplement: Document S2. Article plus Supplemental Information [file mmc2.pdf]

Cell Systems

Article

Modeling Enzyme Processivity Reveals that RNA-Seq Libraries Are Biased in Characteristic and Correctable Ways

Graphical Abstract

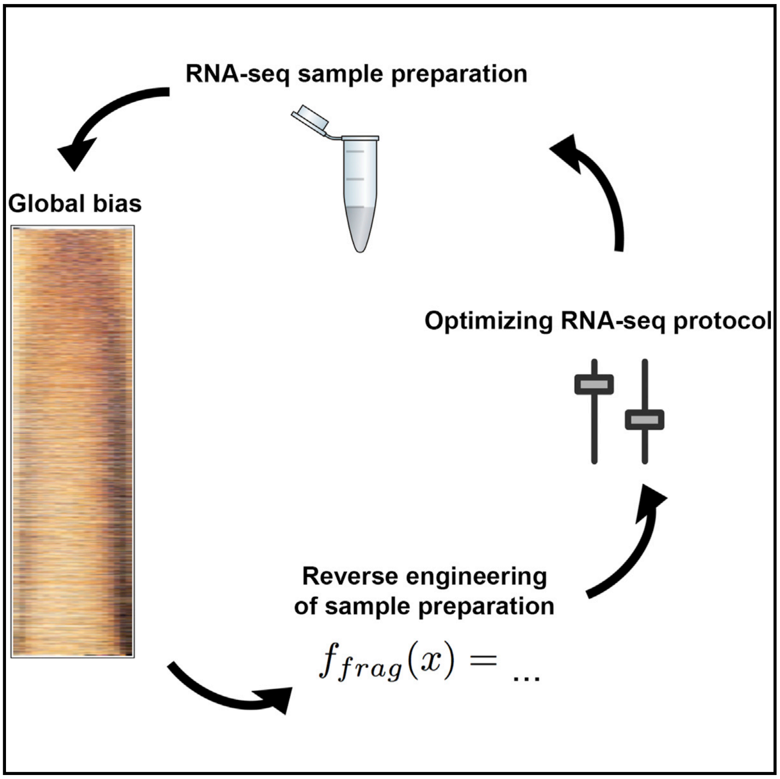

Highlights

- Characterization of global RNA-seq biases specific to library preparation protocols
- Mathematical framework to reverse engineer enzyme reactions that cause bias
- Insights from reverse engineering allow optimization of RNA-seq protocols
- Lowered incubation temperatures during library preparation improve sensitivity

Authors

Nathan Archer, Mark D. Walsh,
Vahid Shahrezaei, Daniel Hebenstreit

Correspondence

v.shahrezaei@imperial.ac.uk (V.S.),
d.hebenstreit@warwick.ac.uk (D.H.)

In Brief

Experimental procedures for preparing RNA-seq and single-cell RNA-seq libraries are based on assumptions regarding their underlying enzymatic reactions. However, the fairness of these assumptions varies between libraries, and most protocols have characteristic biases. Here, we investigate the mechanistic basis of these biases, provide correction factors, and increase the accuracy of transcript quantitation in RNA-seq and scRNA-seq.

Data Resources

GSE84785

Archer et al., 2016, Cell Systems 3, 467–479
November 23, 2016 © 2016 The Author(s). Published by Elsevier Inc.
http://dx.doi.org/10.1016/j.cels.2016.10.012

OPEN ACCESS
CellPress

Modeling Enzyme Processivity Reveals that RNA-Seq Libraries Are Biased in Characteristic and Correctable Ways

Nathan Archer,[1] Mark D. Walsh,[1] Vahid Shahrezaei,[2,*] and Daniel Hebenstreit[1,3,*]
[1]School of Life Sciences, University of Warwick, Coventry CV4 7AL, UK
[2]Department of Mathematics, Imperial College, London SW7 2AZ, UK
[3]Lead Contact
*Correspondence: v.shahrezaei@imperial.ac.uk (V.S.), d.hebenstreit@warwick.ac.uk (D.H.)
http://dx.doi.org/10.1016/j.cels.2016.10.012

SUMMARY

Experimental procedures for preparing RNA-seq and single-cell (sc) RNA-seq libraries are based on assumptions regarding their underlying enzymatic reactions. Here, we show that the fairness of these assumptions varies within libraries: coverage by sequencing reads along and between transcripts exhibits characteristic, protocol-dependent biases. To understand the mechanistic basis of this bias, we present an integrated modeling framework that infers the relationship between enzyme reactions during library preparation and the characteristic coverage patterns observed for different protocols. Analysis of new and existing (sc)RNA-seq data from six different library preparation protocols reveals that polymerase processivity is the mechanistic origin of coverage biases. We apply our framework to demonstrate that lowering incubation temperature increases processivity, yield, and (sc)RNA-seq sensitivity in all protocols. We also provide correction factors based on our model for increasing accuracy of transcript quantification in existing samples prepared at standard temperatures. In total, our findings improve our ability to accurately reflect in vivo transcript abundances in (sc)RNA-seq libraries.

INTRODUCTION

RNA sequencing (RNA-seq) has quickly become the standard method for transcriptomics (Wang et al., 2009) and has been further developed into a number of modified protocols that allow detection from single cells (single-cell RNA-seq [scRNA-seq]) (Tang et al., 2011). The power of scRNA-seq to reveal cell population heterogeneity in transcriptome-wide fashion has made it the focus of intense recent research activity aimed at its further development and on analysis techniques (e.g., Grün et al., 2014; Kim and Marioni, 2013; Nakamura et al., 2015).

The typical workflow of an RNA-seq assay involves the extraction (and often further purification) of mRNA from cells, the preparation of a sequencing library including fragmentation, linear (Hashimshony et al., 2012) or PCR amplification, next-generation sequencing, and computational processing and analysis of the resulting data. Although a great variety of different RNA-seq protocols have been developed, virtually all (except for direct RNA sequencing [Ozsolak and Milos, 2011]) include the basic cDNA production steps of reverse transcription (often referred to as first-strand synthesis) and second-strand synthesis, which often corresponds to an extended first cycle of the subsequent PCR amplification (Figure 1A). The cDNA replaces the less robust RNA with DNA and is required for the introduction of adapters to enable next-generation sequencing, unless special adaptions are used (Gansauge and Meyer, 2013). The enzymes used in cDNA production are processive (Von Hippel et al., 1994) and thus incorporate many nucleotides before the reaction stops. The exact syntheses starting and stopping points are unclear and introduce complex positional dependencies, which are crucial for the resulting RNA-seq coverage (Figure 1B).

Several steps in the library preparation procedures lead to over- and/or under-representation of sequences with regards to the starting material, introducing biases in the RNA-seq quantification. This can be partially experimentally corrected by employing molecular barcodes (Islam et al., 2014), although these have other disadvantages, such as PCR and sequencing errors that bias results (Macosko et al., 2015). Some types of bias, such as non-uniform primer binding (Hansen et al., 2010) or fragmentation efficiency (Griebel et al., 2012; Quail et al., 2008), affect the local coverage within transcripts and can be computationally corrected to a degree. However, the vast majority of (sc) RNA-seq datasets show peculiar *global* shapes, that is an overall pattern concerning transcript coverages that depends on the transcripts' lengths (see below, Results, and glossary for terms we use in Box 1). It was noted before that this is probably due to cDNA production (see below) (Mortazavi et al., 2008). However, the effect remains uncorrected by analysis tools (Stegle et al., 2015) and is not understood, and the systematic bias it introduces is potentially much stronger than local variation.

Since the major goal of RNA-seq is to accurately infer (relative) expression levels or sequence structure of the original mRNAs, these biases are problematic and need to be taken into account. This issue is particularly relevant for scRNA-seq, where absolute transcript quantification is desired and where the bias in coverage by sequencing reads can affect sensitivity. While losses at each step of a standard RNA-seq protocol are uncritical due to a sufficient supply of starting material, they limit chances

This is an open access article under the CC BY license (http://creativecommons.org/licenses/by/4.0/).

OPEN ACCESS
CellPress

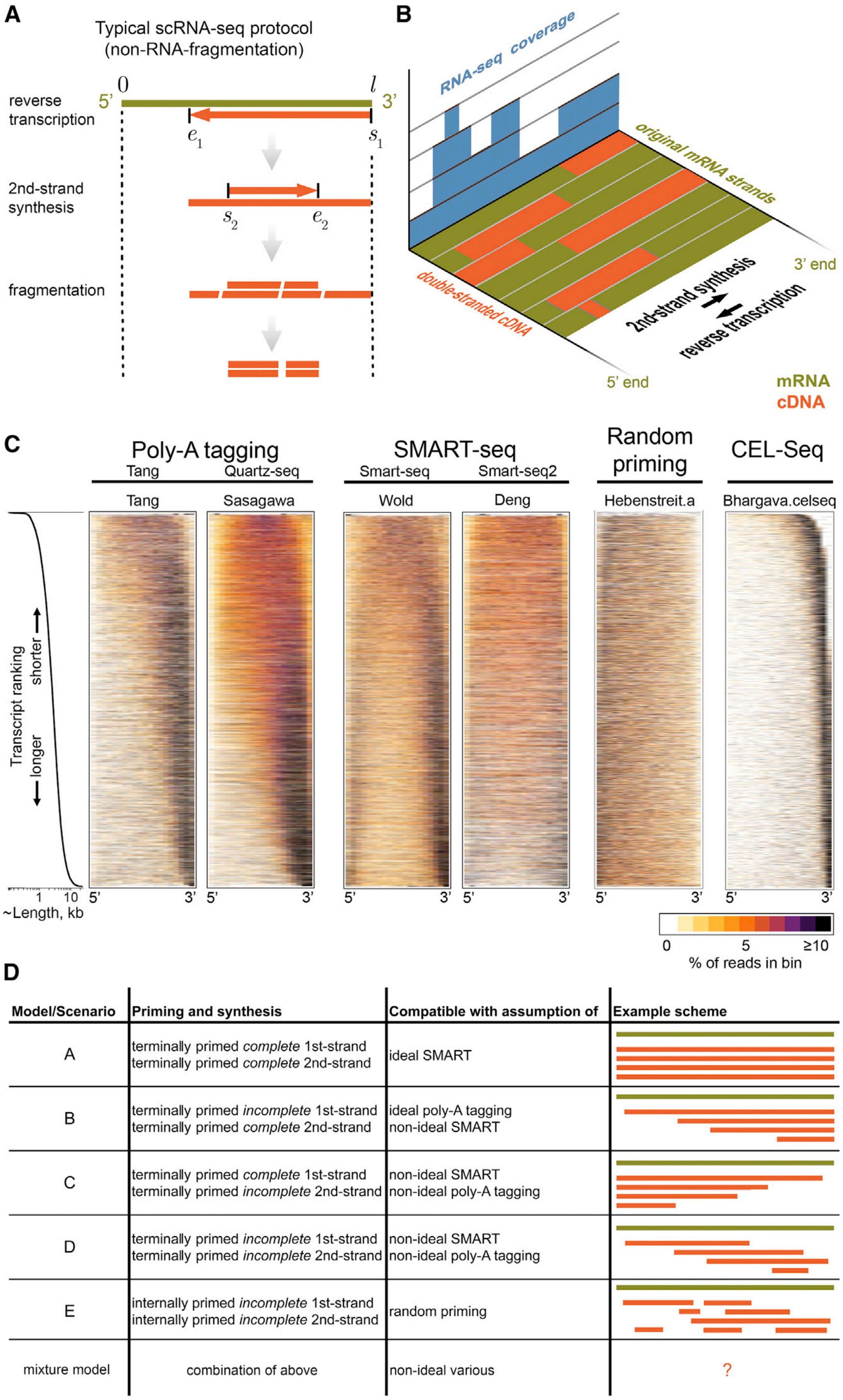

Figure 1. cDNA Conversion Yields Biases of RNA-Seq Coverage

(A) Library preparation for next-generation sequencing involves reverse transcription and second-strand synthesis, followed by fragmentation. Depending on the protocol, reverse-transcription starts and ends at certain points for first-strand synthesis (s_1 and e_1, respectively) and second-strand synthesis (s_2 and e_2).

(B) The original mRNA (olive) is thus often non-uniformly represented by double-stranded cDNA (orange), which biases detection by RNA-seq (blue).

(C) RNA-seq coverage along transcripts for different datasets. Sequencing reads were mapped to murine, non-overlapping RefSeq transcripts without isoforms. All detected transcripts (~10,000) were ordered from shortest (top) to longest (bottom), were adjusted to have identical length, and were divided into 20 bins each. The percentage of reads in each bin is color coded for each transcript (see legend). The distribution of transcript lengths is shown on log scale on the left. This distribution corresponds to the Wold dataset but is representative of the others, subject to minor variations due to different numbers of detected transcripts. Details of the datasets shown are listed in Table 1. Unbiased coverage within transcripts would result in uniformly orange rectangles. More datasets are shown in Figure S1.

(D) Simplified models/scenarios of RNA-seq library preparation outcomes based on priming strategy and synthesis success.

of transcript detection and absolute quantification in scRNA-seq. Ideally, the mass of every single original mRNA should be harnessed as completely as possible for the next-generation sequencing step at the end of an scRNA-seq protocol. To do that, one must understand systematic non-uniformities in scRNA-seq coverage.

In the present work, we introduce an analytical and computational framework that allows "reverse engineering" of reactions and enzyme kinetics during RNA-seq library preparation. Applying this framework, we are able to identify polymerase processivities as the main determinants for the global coverage shapes. Our models also yield correction factors for quantification, which demonstrate that currently used measures are inadequate. The insights into molecular reactions that our framework allows can be further exploited to improve RNA-seq protocols, as we demonstrate experimentally.

RESULTS

Below, we will analyze a selection of RNA-seq strategies, mostly for scRNA-seq, but covering virtually all widely used protocols, and focus on the coverage by sequencing reads along transcripts. The main variation between these protocols concerns the first- and second-strand priming strategies.

The first published scRNA-seq strategy (Tang et al., 2009), which we term the poly-A-tagging protocol, is designed to ligate a second-strand primer to an adenine stretch that is added by terminal transferase to the end of the poly-A tail-primed first-strand. Thus, coverage critically depends on where reverse

Box 1. Glossary

cDNA	Single- or double-stranded DNA obtained from reverse transcription of mRNA, followed by second-strand synthesis (if double stranded).
Conditional probability	$P(x \mid y)$ is the probability of event "x" under the condition that "y" has occurred.
Coverage	The density of sequencing reads aligning to known bases, usually along exons within gene bodies.
First-strand synthesis	Same as reverse transcription. Polymerization of a complementary DNA (cDNA) strand along an mRNA by reverse transcriptase.
FPKM	Fragments per kilobase of transcript per million mapped reads. Widely used measure for expression levels determined by RNA-seq.
Likelihood	A measure for how well a model agrees with the data.
Likelihood ratios	Compares the goodness of fit of two models by calculating the ratio between their likelihoods.
Markov Chain Monte Carlo	Class of methods that allow efficient sampling from a probability distribution and are commonly used to produce estimates of posterior distributions in Bayesian statistics.
MCMC	See Markov Chain Monte Carlo.
Overhang	Unpaired nucleotides resulting in a single-stranded stretch at the 5′ or 3′ end of double-stranded DNA.
Parameters	Values that our models depend on. They determine the exact shapes of the coverage functions and are learned from a specific dataset through the fitting process (see MCMC). The parameters of our models have intuitive interpretations:
	$d + 1$ ratio of fragmentation efficiencies inside strands versus close to ends.
	h distance (bases) from ends over which fragmentation efficiency is reduced.
	$1/\theta_1$ average synthesis length of reverse transcription (processivity).
	$1/\theta_2$ average synthesis length of second-strand synthesis (*processivity*).
	α fraction of PCR-selected full-length strands.
Priming strategy	The method by which reverse transcription or second-strand synthesis is primed to initiate the reaction—including sequence-specific primers, Oligo(dT) primers, random primers, or others.
Processivity	The ability of an enzyme to catalyze consecutive reactions between association and dissociation from its substrate. In our context, we use the term as the average number of nucleotides incorporated (i.e., the synthesized length) in one uninterrupted process (on an infinitely long template).
Reverse transcription	See First-strand synthesis.
Second-strand synthesis	Polymerization of a second DNA strand complementary to the first cDNA strand by a DNA polymerase.

transcription stops. An improved version of this protocol was published as "Quartz-seq" (Sasagawa et al., 2013). By contrast, complete ("full-length") sequencing coverage along the whole mRNA has been a selling point of different library preparation protocols, as it is believed to correspond to more reads per transcript and/or better resolution of splice variants (Picelli et al., 2013; Ramsköld et al., 2012). Particularly successful in this respect is the second scRNA-seq approach we are studying, termed "Switching Mechanism At the 5′ terminus of the RNA Transcript" (SMART) (Zhu et al., 2001). Here, the second-strand primer binds to the overhang generated by the addition of several non-templated cytosines by the reverse transcriptase upon completion of full-length of the first-strand, which is primed from the poly-A tail. SMART-based scRNA-seq, and its variants (e.g., "Smart-seq2"), has become a de facto standard (Deng et al., 2014; Islam et al., 2012; Picelli et al., 2013; Ramsköld et al., 2012; Shalek et al., 2013). Both poly-A-tagging and SMART protocols are usually subjected to variable numbers of PCR cycles. An extended first PCR cycle is used to synthesize the second-strand, while later cycles also enrich *complete* second strands by using primers flanking the 3′ ends of first-strands.

While the bulk of our analysis will be devoted to methods derived from poly-A tagging and SMART, we will also briefly discuss the linear-amplification-based scRNA-seq strategy CEL-seq (Hashimshony et al., 2012, 2016). CEL-seq compares unfavorably to the above scRNA-seq protocols in some studies in terms of its technical variation (Bhargava et al., 2014) and is based on a complex sequence of enzymatic conversions; the mRNAs are reverse transcribed based on poly-A priming using molecular barcode containing primers, followed by random-primed second-strand synthesis, in vitro transcription, RNA fragmentation, and another round of first- and second-strand syntheses. Finally, only fragments containing the 3′ end with regards to the original mRNA are selected by PCR. Inference of expression levels is based on counting these fragments and/or unique barcodes, while coverage along transcripts is ignored. CEL-seq thus follows a different principle than the other protocols.

In addition to these single-cell techniques, we include two bulk methods for comparison. First, we analyze the classical RT-PCR/RNA-seq protocol based on random-oligonucleotide primed first-strand synthesis, followed by randomly primed second-strand synthesis based on RNaseH-nicking (CSHL, 2005) (with fragmentation after cDNA production). This priming strategy is not common in scRNA-seq, as the usage of 3′ poly-A tail binding primers reduces priming of rRNA, thus making purification of mRNA unnecessary and potentially reducing losses of the limiting starting material. However, it provides a useful

comparison because it gives rise to very different coverages as the above protocols and is still commonly employed for qPCR. Second, we include an RNA-fragmentation-based dataset (Vahedi et al., 2012), which fragments mRNA instead of cDNA and thus strongly reduces the coverage bias due to cDNA-production. While this is routinely applied in standard RNA-seq, it is not used for scRNA-seq, presumably for fear of degrading and losing mRNA and because it precludes direct poly-A priming. This allows us to compare above protocols to a popular and potentially bias-free one.

The principles of the above mentioned RNA-seq protocols are mostly based on assumptions, and it is unclear how closely these reflect the experimental reality. It has been pointed out before, for instance, that SMART protocols may increase the portion of full-length products in the final reaction mixture by excluding incomplete first-strand synthesis products (due to reduced efficiency of the SMART mechanism inside the mRNA compared to its end), rather than by improving or completing their synthesis (Hebenstreit, 2012; Shapiro et al., 2013). We wanted to explore from a general and quantitative perspective how reliable the above assumptions are and what trade-offs between complete coverage, loss of starting material, and position bias are to be expected for the various protocols.

Thus, we visualized the sequencing read distributions in actual datasets generated by a variety of RNA-seq and scRNA-seq library preparation protocols. We group protocols into poly-A tagging-like, SMART-like, random priming, CEL-seq, and RNA fragmentation (Table 1). To limit the influence of confounding factors in our analyses, we selected datasets for a single species only (mouse) and mapped reads to non-overlapping RefSeq transcripts without splice variants as it was done before (Li et al., 2010b); overlapping genes and genes with multiple isoform annotations would potentially give rise to more complex coverage shapes that are independent from the protocol-specific effects we want to study.

In order to effectively visualize coverage and define global shapes present within each dataset, we ordered transcripts according to their lengths and color-coded read densities in 20 bins along the transcripts after length normalization (Figure 1C). This highlights the "noisiness" of the data due to the various bias sources but also confirms some previous observations: poly-A primed libraries tend to exhibit a 3′ bias (Mortazavi et al., 2008), SMART protocols produce reasonable coverage even for longer transcripts (Ramsköld et al., 2012), and the profiles depend on transcript length (Bohnert and Rätsch, 2010) (Figure 1C). Random priming yields more uniform, yet 5′-biased coverage, as previously reported (Mortazavi et al., 2008) (Figure 1C). Virtually all datasets feature underrepresented regions close to transcript ends, presumably due to inefficient fragmentation as discussed above. We include a plot for CEL-seq data, which confirms selection of 3′ fragments (Figure 1C).

Several features of the data have been noted before (Adiconis et al., 2013; Ramsköld et al., 2012) but warrant more discussion and analysis: the 3′ bias in the SMART and poly-A-tagging datasets tends to worsen with increasing transcript lengths, whereas the coverage of shorter transcripts is more uniform and even 5′ biased in some cases. In addition, bimodality in the coverage (high read densities at 5′ and 3′ ends, low density in the centers of transcripts) appears for transcripts of intermediate and/or long lengths (> ~3 kb) in most SMART-seq datasets (Figure 1C; more datasets are shown in Figure S1). It is also noteworthy how similar these aspects are among poly-A-tagging and SMART protocols, given the differences between these. Although the graininess of the data is affected by the amount of starting material/PCR cycles, the bias shapes appear independent of this (Bhargava datasets, Figure S1). The only protocol without strong systematic bias (aside from underrepresented ends) is RNA fragmentation (Figure S1).

As a first step toward understanding these phenomena, we asked whether they could be recapitulated by simplified models (Figure 1D). To this end, we defined the expected and assumed differences among the protocols, including their possible limitations, in a set of five abstracted and simplified models (these are summarized graphically in Figure 1D). We label these from "A" to "E," which roughly increase in complexity, starting with the idealistic scenario of full-length syntheses for both first- and second-strand "A." This would be compatible with an optimally functioning SMART protocol, free from any coverage bias, similar also to earlier assumptions of uniform coverage of RNA-seq data (using the measure of fragments per kilobase per million total fragments [FPKM]; see below). Models B and C correspond to successful full-length selection for fragments containing either the 3′ or 5′ transcript end, respectively (i.e., by PCR with 3′ flanking primers or full-length SMART on 5′ end, respectively). We consider models that abstract non-full-length poly-A tagging (model D) and random priming (model E) and also the possibility of a combination of these simpler models (Figure 1D). The models are discussed in greater conceptual detail below.

RNA-seq library preparation can be understood as a stochastic process, where steps in the protocol depend on preceding ones and are associated with varying degrees of randomness. A convenient and very intuitive way to model this is by using conditional probabilities (Box 1). For instance, given that first-strand synthesis starts at position s_1 along the transcript, it might end at position e_1 with probability $P(e_1 \mid s_1)$. The starting position of the second-strand synthesis, s_2, would then depend on this, giving $P(s_2 \mid e_1)$, and so forth (Figure 1A, see Method Details for details). We use this approach to capture the various aspects of the protocols with the aim of quantitatively and formally understanding their expected influence on shaping the distributions of sequencing read starting positions (mathematical models can be found in the Models section of Method Details). While our framework is very flexible and allows us to easily include several different factors, we focused on the effects of enzyme reactions during cDNA conversion as captured by our minimal models A to E (Figure 1D). We thus do not consider sequence-specific biases and the lengths of primers. We also exclude factors that are expected to cause overall loss with regards to the starting material but do not introduce bias. Failed poly-A tail priming, for instance, will probably affect different transcripts with roughly equal probability, so we do not consider it in our analysis. In contrast, usage of random first-strand primers plausibly will favor cDNA conversion of longer transcripts, as the chances of binding are higher.

Depending on the protocol, the start and endpoints of enzymatic syntheses during cDNA conversion are determined not only by priming positions but also by the enzymes' average

Table 1. Previously Published Datasets Used in This Study

Group	Name/Reference	Accession Number	Sample	Library Protocol	Read Type	Read Number	Reads Mapped (%)
poly-A tagging	Tang et al. (2009)	GSM365014	single cell, oocyte	Tang	50 bases SE, SOLiD	25M	36
	Sasagawa et al. (2013)	GSM1036495	50 cells, embryonic stem cells	Quartz-seq	102 bases PE, Illumina	85M	96
	Wei et al. (2011)	GSM523211	> μg, resting Th2 cells	Tang	36 bases SE, Illumina	11M	57
SMART	Deng.smartseq (Deng et al., 2014)	GSM1112540	single cell, 4-cell stage embryo	Smart-seq	53 bases SE, Illumina	27M	42
	Bhargava.AA.ng	GSM1231200	1 ng	Smart-seq	99 bases SE, Illumina	25M	50
	Bhargava.SFM.ng	GSM1231198	1 ng			24M	41
	Bhargava.AA.pg	GSM1231212	25 pg			23M	51
	Bhargava.SFM.pg (Bhargava et al., 2014)	GSM1231210	25 pg mRNA, embryoid bodies			24M	41
	Wold (ENCODE project)	ENCSR814JMM	50 cells, cerebellar granule layer	Smart-seq	100 bases SE, Illumina	48M	26
	Deng.smartseq2 (Deng et al., 2014)	GSM1278036	single cell, fibroblast	Smart-seq2	43 bases SE, Illumina	28M	42
	Mahata et al. (2014)	ERR489030	single cell, activated Th2 cells	Smart-seq	75 bases PE, Illumina	23M	23
Random priming	Hebenstreit.a	GSM710184	> μg, resting Th2 cells	Random priming, RNaseH	36 bases	16M	46
	Hebenstreit.b (Hebenstreit et al., 2011)	GSM710183			41 bases SE, Illumina	26M	37
CEL-seq	Bhargava.celseq (Bhargava et al., 2014)	GSM1231230	1 ng mRNA, embryoid bodies	CEL-seq	100 bases PE, Illumina (3′ read used only)	30M	16
	Celseq2 (Hashimshony et al., 2016)	GSM2076520	single cell, fibroblast	CEL-seq2	35 bases (trimmed) PE, Illumina (3′ read used only)	0.65M	40
RNA fragmentation	Vahedi et al. (2012)	GSM994539	> μg, resting Th2 cells	TruSeq	100 bases SE, Illumina	40M	46

SE, single end; PE, paired end.

OPEN ACCESS CellPress

Table 2. Theoretical Expectations, Corresponding to Likelihoods, of Transcript Coverage with Different Models

Model/ Scenario	Coverage Function $f(x, l, \ldots) =$
A	$e^{-l(\theta_1+\theta_2)}$
B	$\frac{1}{(\theta_1+\theta_2)}\left[\theta_1 e^{-2l(\theta_1+\theta_2)+(\theta_1+\theta_2)(l+x)} + \theta_2 e^{-l(\theta_1+\theta_2)}\right]$
C	$e^{-\theta_1 l-\theta_2 x}$
D	$\frac{1}{(\theta_1+\theta_2)}\left[\theta_1 e^{-\theta_1(l-x)} + \theta_2 e^{-\theta_1 l-\theta_2 x}\right]$
E	$\frac{\alpha_1\alpha_2}{\theta'_1(\theta'_1+\theta'_2)}\left[l - \frac{1}{\theta'_1+\theta'_2} - \frac{1}{\theta'_1} - \frac{\theta'_1 e^{-l(\theta'_1+\theta'_2)}}{\theta'_2(\theta'_1+\theta'_2)} + \frac{(\theta'_1+\theta'_2)e^{-l\theta'_1}}{\theta'_1\theta'_2}\right]$

Theoretical expectations (likelihood) of transcript coverage with different models (Figure 1D). x is the absolute position within the transcript ($x = 0$ at the 5′ end), l is the absolute transcript length (Figure 1A), and θ_1 and θ_2 are the inverse processivities of first- and second-strand syntheses, respectively. θ'_1 and θ'_2 are modified processivities for model E (see Method Details). α_1 and α_2 are the probabilities of first- and second-strand priming at a certain position, respectively. The fragmentation-related terms and parameters (d, h) are omitted for clarity.

synthesis lengths, their "processivities." The enzymes' processivities are in general likely to depend on several parameters, such as temperature or nucleotide concentration, and could reflect eventual stops in the synthesis process or physical detachments of the enzyme from its template or both. The processivity of the reverse transcription is influenced by mRNA secondary structure as well, which again depends on other factors, including sequence and temperature (Joseph and David, 2001). In total, cDNA strand synthesis length is most commonly assumed to roughly follow geometric/exponential distributions (Bibillo and Eickbush, 2002; Von Hippel et al., 1994). We adapt this for our model and assume $P(e_1 \mid s_1)$ and $P(e_2 \mid s_2)$ follow exponential distributions, taking also account of possible full-length synthesis (see Figure S2 and STAR Methods). The distributions are subject to parameters θ_1 and θ_2, which are inversely proportional to the processivities of the first- and second-strand synthesis. Finally, we include terms in our model to account for reduced fragmentation efficiency at the ends of the double-stranded cDNAs in the library. This has the form of a step change of sequencing probability as given by parameter d over distance h from either end.

Based on these considerations, we derived expressions for the expected coverage of our models A to E as functions of transcript length l. These have the forms of various combinations of exponential terms and are of moderate complexity (Table 2, shown without d, h terms for clarity). It is of note that the expression for model A and its notion of "ideal" SMART can be interpreted in two ways; either full-length syntheses are achieved by very high processivities (giving essentially $\theta_1 = \theta_2 = 0$), or full-length cDNA is enriched over incomplete products (e.g., by PCR and/or the SMART mechanism), allowing for higher θ_1 and θ_2, but implying exponentially decreasing sequencing efficiency with increasing mRNA length.

Models A, B, and C restrict the global coverage shapes that can be expected to a straight line or simple exponential decreases from either side, respectively, for all lengths (Figure 2A). However, the coverage obtained with model "D" under realistic parameter settings resembles the experimental SMART datasets, capturing the transition from 5′ to bimodal to 3′ bias (Figure 2A). Model D also predicts lower densities for the 5′ edges compared to the 3′ edges throughout, in the regions where fragmentation efficiency is reduced (Figure 2A). This too appears to mirror the experimental data for all relevant protocols. Using the same parameter settings, we obtain equally promising shapes for model E, which resembles the 5′-biased random-priming data it is designed to explain (Figure 1C). The overall characteristics of the bias shapes are conserved if the skewed distribution of natural transcript lengths is replaced with a linear function (Figure S3a).

We proceeded to test fits of our models to the actual datasets and infer parameters using a Markov Chain Monte Carlo (MCMC) approach (STAR Methods). As the coverage in models A and C does not depend on all the parameters, in these cases a subset of parameters are inferred. We compare the quality of the fits we obtain for all models based on their likelihoods (STAR Methods), which reveals that models B and D provide best fits for poly-A-tagging and SMART datasets (Figure S3b). Model D, in particular, captures well the changing coverage shapes with increasing transcript lengths (Figure 2B). Given these findings, we presumed that second-strand priming and partial PCR selection of both, poly-A-tagging and SMART protocols is captured best by a combination of models B and D, which indeed yields the best fits (Figures S3b and 2B). The parameter values we obtained for the combined model suggest that the average synthesis lengths for the first- and second-strands are about 5–10 and 1–3 kb, respectively (Figures 2C and S4), which agrees with estimates from the literature (Joseph and David, 2001). The parameterized models capture behavior observed in in vivo datasets, suggesting that the assumptions made during modeling are reasonably conservative. For example, increasing the number of PCR cycles used in the SMART protocol should result in an increase in the proportion of the (full-length second-strand) model B over D, as parameterized by parameter α (Method Details). The Bhargava dataset allows testing this as it includes samples subjected to different numbers of PCR cycles. α indeed increases significantly ($p < 10^{-9}$, one-sided Mann-Whitney U test) with higher numbers of PCR cycles for two different biological samples (Activin A treated [AA]; serum-free media [SFM]; Figure 2D).

Our parameterized models allow us to test the common assumptions about how scRNA-seq and RNA-seq protocols work. Model A and model C clearly perform worse as these restrict the coverages to patterns that are not observed in the data, which is reflected in the goodness-of-fit statistics (Figure S3b). This suggests that the common assumptions regarding SMART protocols are too optimistic. For example, second-strand synthesis appears to frequently start within transcripts, not at ends only, and selection for complete second-strands is imperfect, which explains the similarities between poly-A-tagging and SMART protocols. A similar observation termed "strand invasion" was made for the nanoCAGE technique recently, where it was found that the second-strand primer ("template switching oligo") can bind the first-strand internally at complementary sequences (Tang et al., 2013). As expected, model E fits well the random-priming datasets, but

OPEN ACCESS
CellPress

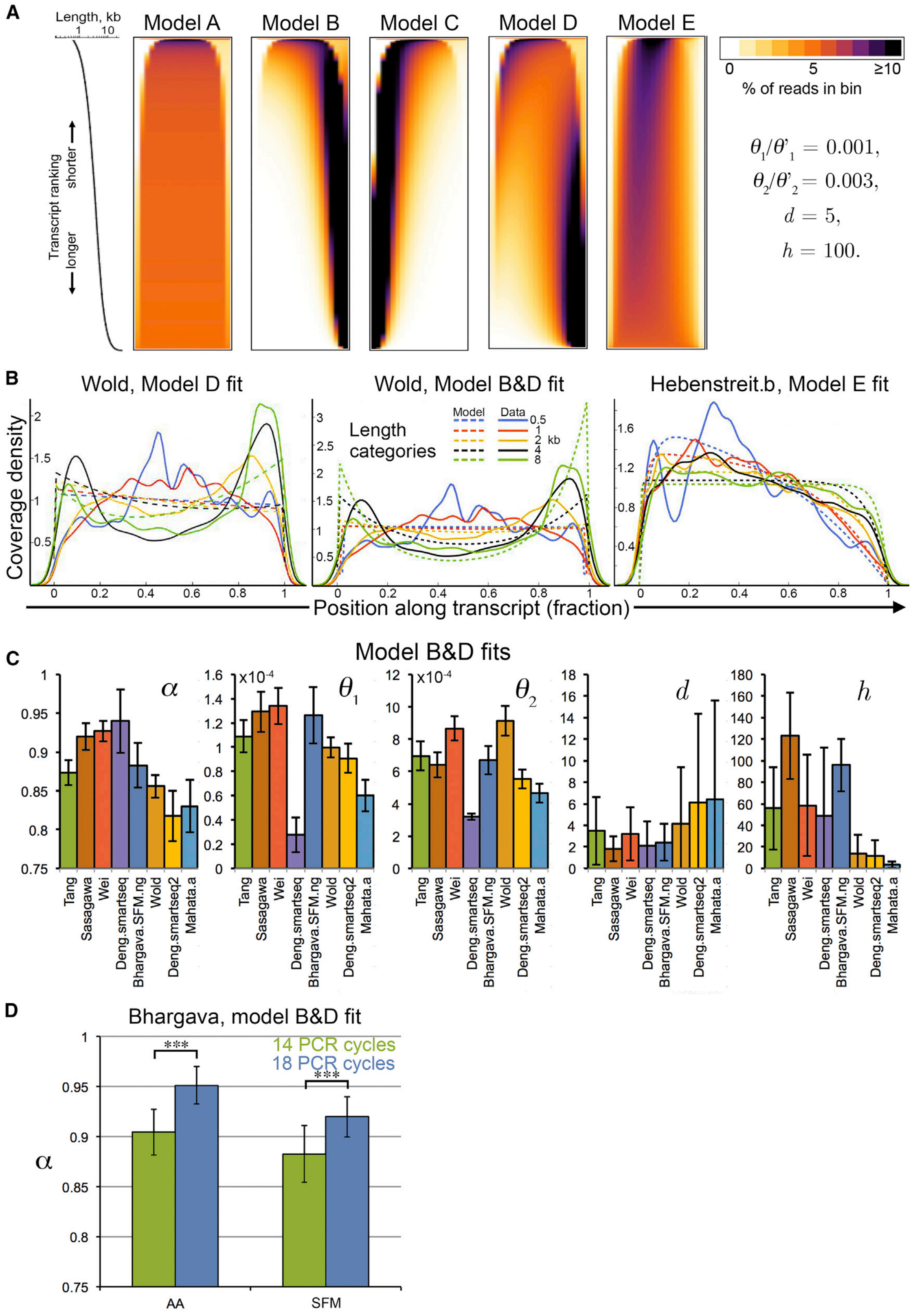

(legend on next page)

not the poly-A-tagging and SMART datasets (Figures 2B and S3b; see Figure S5 for parameter estimates of the remaining models).

Altered Incubation Temperature and Model-Driven Improvement to RNA-Seq Protocols

While the goodness of our fits and the underlying logic suggest our modeling approach is valid, we sought further experimental confirmation. To this end, we prepared RNA-seq samples designed to specifically perturb single parameter values only and sequenced them on an Illumina MiSeq sequencing machine. We focused on incubation temperature for enzyme reactions because it is both experimentally accessible and interpretable: we reckoned that it should affect polymerase processivity. We prepared libraries using lowered temperatures during reverse transcription (25°C instead of the standard 42°C) and/or second-strand synthesis (42°C instead of the standard 72°C); protocols were based on and generated by SMART-seq or Quartz-seq and began with different starting RNAs (poly-A+, total RNA, single cell; see Table S1 for a list of all samples).

If the notions underlying our modeling approach are correct, the changed temperatures should change the corresponding parameter estimates while the remaining parameter estimates should remain the same. The parameter estimates we obtained confirm this reasoning; lowering first-strand temperature changes θ_1 estimates without affecting θ_2 significantly, and vice versa if second-strand temperature is changed (Figures 3A and 3B; examples for coverage plots Figure S6). An exception is the significantly different θ_1 estimate upon changing second-strand temperature with first-strand synthesis at 25°C; however, in this case, the median is very close.

We note that estimation of θ_2 for SMART-seq is less precise, as the PCR step with flanking primers means that the original second-strand contributes substantially less to the shape of the coverage (compare this to model A, "ideal SMART-seq," which would not even allow estimation of θ_1, as discussed above), thus obscuring the temperature-related differences. For this reason, we excluded SMART-seq samples from the plot for θ_2 in Figure 3B (they are shown as Figure S7a) and instead add SMART-seq samples where we omitted the PCR step (Figures 3A and 3B), which yields similar results as the other protocols.

This analysis also revealed an unexpected feature of cDNA synthesis: lowering temperatures appears to *increase* processivities of the enzymes (Figures 3A and 3B). This observation suggests that lowering incubation temperatures should improve the yield of RNA-seq protocols. We therefore measured by Qubit the absolute amount of cDNA produced from the same starting amounts of mRNA and synthesis reactions carried out at the temperatures described above. We observed significantly ($p \leq 0.001$, one-sided t test) increased cDNA synthesis at lower temperatures, with an optimum yield upon lowering both temperatures (Figure 3C). To further investigate the increase in processivity, we turned to RNA-seq again. We prepared RNA-seq samples from the same RNA, aliquoted the samples for each temperature, and sequenced these on the same lane using indexed primers. Increased processivities should increase the proportion of longer mRNAs within the samples. We thus compared the relative representation by sequencing reads of transcripts of different lengths normalized to corresponding read numbers at the standard incubation temperatures. Indeed, we observe significantly increased representation of longer transcripts (Figure 3D); this is in accordance with the Qubit measurement and suggests that polymerase processivity increases at low temperatures. We further compared the numbers of genes detected and the overall numbers of sequencing reads we obtained for transcripts and for spike-in probes, which we had added to a subset of starting RNAs for our RNA-seq samples. This confirms increased yields upon reduced temperatures (Figures 3E–3G). Accordingly, RNA-seq also became more sensitive: we were able to detect lower concentrations of the spike-in probes (Figure 3H). Notably, reducing the incubation temperatures does not appear to increase local bias. The coverage heatmaps do not exhibit obvious visual differences regarding their "noisiness" (Figure S6), and quantifying this rather suggests improved coverage uniformity upon reduced incubation temperatures (Figure S7b). These findings show an improved RNA-seq performance at lower temperatures and illustrate how insights generated by our framework can be exploited to optimize protocols.

Our framework can also improve analysis of existing data. For example, accurate quantitation of mRNA expression necessitates a thorough understanding of the expected numbers of sequencing reads for different types of transcripts. The simplest notion of linear scaling with transcript length is embodied in the classical RNA-seq FPKM measure, which is now generally understood to be an oversimplification but still widely used. Several approaches to take account of non-uniform read distributions along transcripts have been published and are included in common RNA-seq analysis software, such as CuffLinks (Roberts et al., 2011; Trapnell et al., 2012) or RNA-Seq by Expectation Maximization (RSEM; Li and Dewey, 2011). These approaches focus mostly on the correction of biases *within* transcripts to yield corrected FPKM. Thus, while approaches like these improve isoform quantitation, they do not account for the non-linear scaling of expected read numbers *across* transcripts of

Figure 2. Analysis and Fitting of Models

(A) Coverage heatmaps as in Figure 1C for theoretical models A–E (Table 2), using parameter settings as indicated on the right side. The transcript length distribution of the Wold dataset was used.

(B) Overlays of best fitting models (dashed lines) after Markov Chain Monte Carlo (MCMC) parameter estimation for three different models (D, B&D, and E; left, middle, and right, respectively) for two datasets (Wold, left and middle; Hebenstreit.a, right). Solid lines are kernel density estimates for sequencing read starting positions for different length categories (color code, inset), each containing data for all mRNAs with lengths within 10% of the length category.

(C) MCMC parameter estimates for a selection of SMART and poly-A-tagging datasets. The bar heights correspond to the medians; the error bars correspond to the median absolute deviations.

(D) MCMC parameter estimates for α for four datasets, Bhargava.AA/SMF.ng/pg. AA (Activin A) and SFM (serum-free media) are two different biological samples, which were subjected to 14 or 18 PCR cycles during library preparation as indicated. The bar heights correspond to the medians; the error bars correspond to the median absolute deviations. ***$p < 10^{-9}$, Mann-Whitney U test.

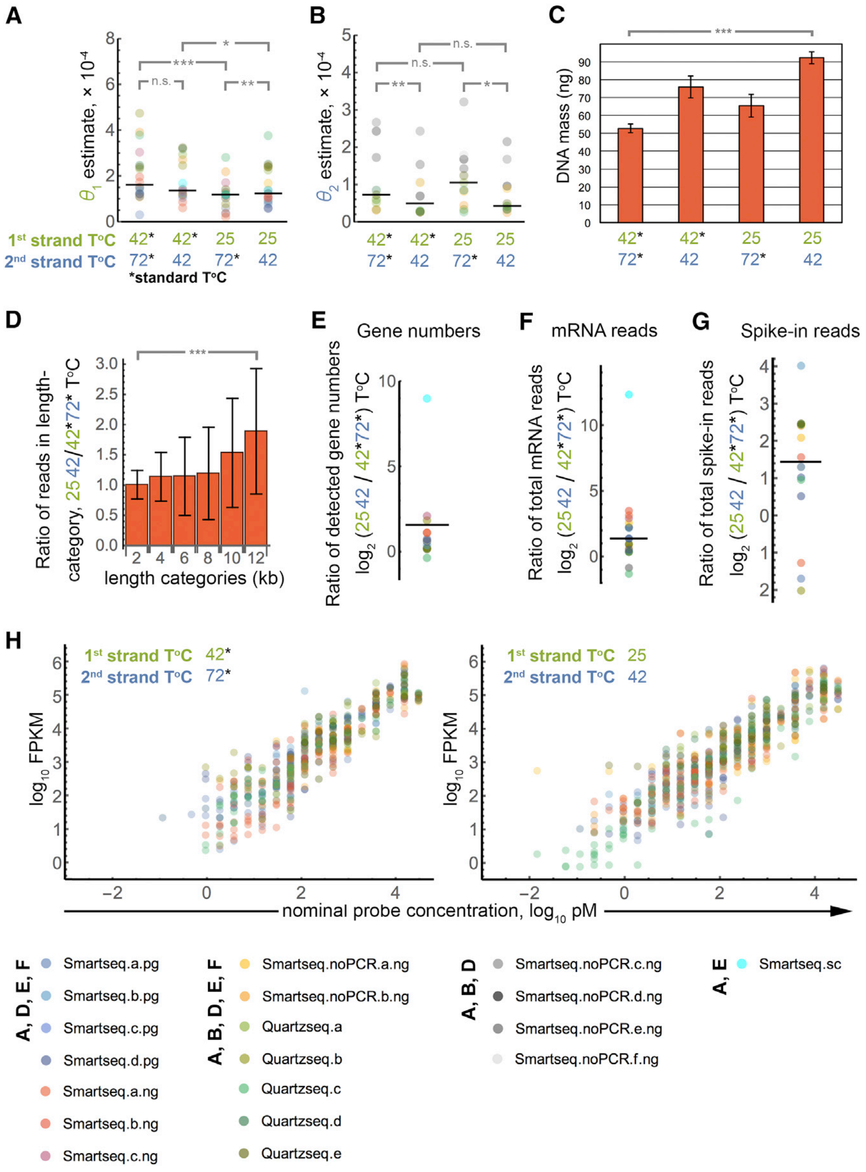

Figure 3. Model Correctly Infers Increased Enzyme Processivities upon Lowered Reaction Temperatures

(A) MCMC parameter estimates for θ_1 for diverse RNA-seq samples (Table S1, color code on bottom of figure) prepared with altered reaction temperatures during first- and second-strand syntheses. "Standard" temperatures were 42°C and 72°C for first- and second-strand, respectively, which were lowered to 25°C and 42°C, respectively, in the designated samples (black horizontal lines indicate the median; *$p \leq 0.051$, **$p \leq 0.01$, ***$p \leq 0.001$, one-sided Wilcoxon signed-rank test).

(B) As (A) for θ_2.

(C) cDNA yield increases upon altered reaction temperatures. Starting amounts were 100 ng mRNA in all samples. DNA mass was measured by Qubit (which does not detect RNA). ***$p \leq 0.001$, one-sided t test.

(D) Fraction of reads mapping to transcripts in different length categories (0–2 kb, 2–4 kb, …, 10–12 kb; >12 kb not included in figure) were determined for sequencing samples with lowered incubation temperatures (first- and second-strand synthesis at 25°C and 42°C, respectively) as indicated by color code at the bottom of the figure. The fractions were then normalized to the corresponding length category for standard incubation temperatures. ***$p \leq 0.001$, Mann-Whitney U test.

(E) Increased numbers of genes are detected upon lowered incubation temperatures. The $\log_2$ ratio of the numbers of detected genes for reduced temperature versus standard temperatures is shown for different RNA-seq samples as indicated by color code at the bottom. The black horizontal line indicates the median.

(F) Increased total sequencing read numbers map to mRNAs upon lowered incubation temperatures. The $\log_2$ ratio of read numbers for reduced temperature versus standard temperatures for different RNA-seq samples as indicated by color code at the bottom is shown. The black horizontal line indicates the median.

(G) Same as (F) for ERCC-mix1 spike-in probes.

(H) Detection of ERCC-mix1 spike-in probes versus nominal concentrations for diverse sequencing samples (color code at bottom). Bar heights in bar charts corresponds to the mean of independent replicates (three in C). Error bars correspond to sample SD.

different lengths (see Note - previous approaches to correct coverage bias in Method Details).

Using our probabilistic framework, we can predict the expected sequencing read numbers for any transcript length, tailored to the library preparation protocol that was used and with parameters inferred from the fit to the corresponding RNA-seq dataset. The expected read numbers are proportional to the areas under the coverage curves that our models predict. Normalizing read numbers of transcripts by the area under the coverage curves for their corresponding length will thus remove the bias and provide a more precise abundance estimate (Method Details). Plotting transcript lengths versus these abundance estimates reveals that model E approaches a linear measure equivalent to FPKM (and model A with $\theta_1 = \theta_2 = 0$) for longer transcripts, while the other models reach plateaus at roughly 4 kb using a single parameter set close to inferred ones (Figure 4A). This highlights protocol-specific gene length dependent sensitivity, since the areas our models predict estimate the relative mass of an mRNA that is converted to double-stranded cDNA, if non-enzyme-induced losses, such as incomplete primer binding, are ignored. Increasing transcript length beyond ~4 kb does not increase sensitivity for non-random priming protocols.

The length scaling of models B–D also means that FPKM-based measures strongly underestimate long transcripts prepared with SMART or poly-A-tagging datasets due to enzyme drop-offs. Benchmarking this prediction based on correlation with standard qPCR or microarray data is not useful per se as

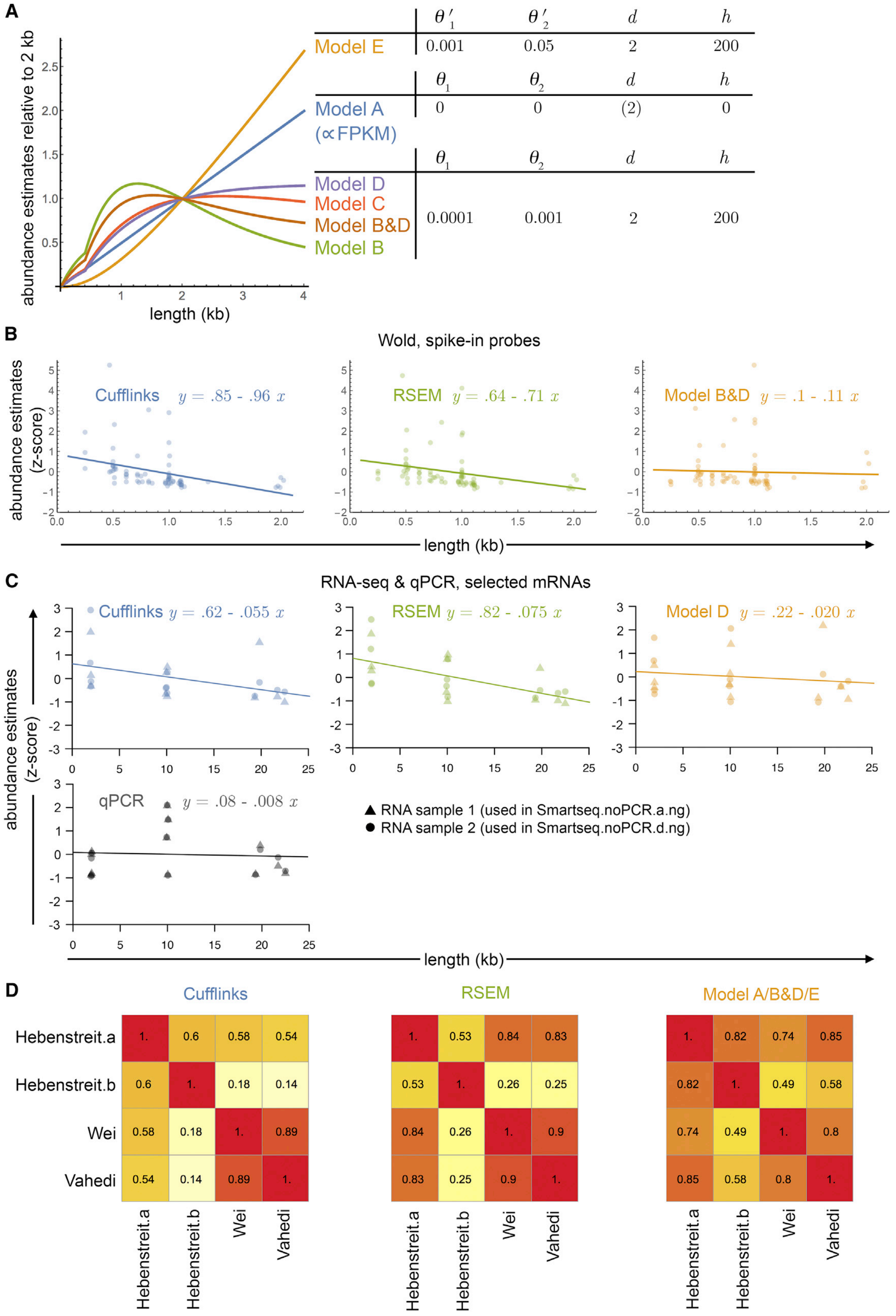

(legend on next page)

the latter are mostly subject to the same protocol-derived biases we are describing. However, the Wold (SMART) dataset contains a set of spike-in RNA probes of different lengths at known relative concentrations (Jiang et al., 2011). The longest of these probes is still comparatively short, at 2,022 bases. Yet, plotting normalized (by nominal probe concentration) and standardized (subtraction of mean and division by SD) Cufflinks or RSEM abundance estimates versus probe length reveals negative trends, confirming our prediction of length-dependent underestimation (Figure 4B). Applying our abundance estimates derived from the fits to the model B&D almost completely eliminates this trend (Figure 4B).

To extend this analysis to greater transcript lengths, we randomly selected 12 genes (Table S2) covering mRNA lengths from $\sim$2 to $\sim$22 kb with intermediate read counts from our RNA-seq samples (Table S1; we included samples corresponding to different incubation temperatures and pooled these after quantifying them separately to maximize the data we were using; the different temperatures are thus not relevant in this analysis). Quantifying their expression levels based on Cufflinks, RSEM, or our Model D fits yields similar results as above; Cufflinks and RSEM estimates exhibit anti-correlation with mRNA length, which is much less pronounced with our model (Figure 4C).

Next, we experimentally test the actual expression levels of the selected transcripts and how they related to their lengths. To this end, we performed qPCR on the same RNA samples, but using primers pairs close ($< \sim$200 bases) to the 3′ ends of the mRNAs in order to strongly limit the effects of enzyme conversion. Indeed, the results demonstrate better agreement with our models than established methods regarding the measured mRNA abundances versus their lengths (Figure 4C). Furthermore, correlation between the qPCR results and RNA-seq expression estimates is higher for our model than for Cufflinks or RSEM (Figure S8a). Notably, transcript expression levels, in particular at the low range, still appeared moderately anti-correlated with length after correction, even with the RNA fragmentation (Figure S8b) and qPCR data (Figure 4C), which support the notion that, biologically, longer genes are on average expressed at a lower level.

Finally, we wanted to test whether the improved, protocol-specific mRNA quantification of our method can reduce library-preparation-induced differences among biological datasets. We have included in our study four datasets for the same cell type (Th2 cells) but using different library preparation protocols. If our method provides more reliable estimates for expression levels, its application should increase correlations among the datasets. We performed individual model fits tailored to the library preparation protocols used in each case and calculated correlations among the datasets with regards to expression levels of all genes. Our models indeed yield higher correlations in most cases compared to Cufflinks- and RSEM-based expression quantification, even though our models do not even take account of sequence-specific, local bias (Figure 4D).

DISCUSSION

We present here a mathematical framework to model library preparation protocols, which addresses several important issues with regards to RNA-seq and, specifically, scRNA-seq. Our approach offers a unified treatment of coverage bias, inference of reaction mechanisms, quantification, sensitivity, and design guidance for library preparation protocols. It can be easily adapted to protocols not covered here and to future developments.

Protocols with limited bias, such as those using RNA fragmentation, will profit from our approach as well; sample preparation of these can potentially be improved based on general insights generated with our framework, such as the cDNA-yield increasing effects of altered incubation temperatures. Furthermore, our models can be easily extended to take account of RNA degradation (see Note - previous approaches to correct coverage bias in Method Details); although this is not implemented yet, our approach of modeling the logics of sample preparation permits inclusion of RNA degradation mechanisms in our models and thus potentially allows analysis and/or correction of their effects. It is also straightforward to integrate and combine our bias correction with previous methods focused on other issues such as the sequence-specific bias; our abundance estimates can be simply included as correction factors, e.g., in the likelihood functions for read numbers of individual isoforms, while the model fitting and thus learning of parameters can be performed on a reduced gene set without overlapping genes.

A first application of our modeling framework suggests an incomplete understanding of the mechanisms underlying SMART protocols and yields processivities of enzymes during library preparation. Notably, our approach presents a novel alternative to processivity measurements by radioactive footprint assays (Bambara et al., 1995). An interesting extension of this aspect of our work would be to explore alternatives to the exponential enzyme drop-offs we assumed. Usage of enzymes under saturating conditions might feasibly result in strands that correspond to concatenates of several individual polymerization processes, requiring more complex models (Method Details).

Another prediction from our study is the substantial underestimation of expression levels of long transcripts with poly-A

Figure 4. Quantification Based on Our Novel Modeling Approach

(A) Length dependency of abundance estimates for our models. The estimates are normalized to unity at length 2 kb. Parameter settings are shown in the table on the right.

(B) Abundance estimates for RNA spike-ins (ERCC-mix1) of the Wold dataset are plotted versus their lengths for three different bias-correction approaches (Cufflinks, blue; RSEM, green; our model B&D fit, orange). Each dot corresponds to one probe. Fitted trend lines and their formulas are shown. Standardized measures (*Z* scores) are used to make the approaches comparable.

(C) Abundance estimates for twelve randomly selected mRNAs (Table S2) covering a wide range of lengths. RNA-seq samples deriving from two different RNA samples (Table S1) were quantified by RSEM, Cufflinks, or our model D as indicated. In parallel, the corresponding two RNA samples (dots and triangle symbols, respectively) were subjected to qPCR for the same twelve genes. Presentation and analysis as in (B).

(D) Correlation matrices for abundance estimates of four datasets for the same cell type (resting Th2 cells) prepared with different library preparation protocols (Hebenstreit.a/b, random priming; Wei, poly-A tagging; Vahedi, RNA fragmentation). Different quantification approaches were used: Cufflinks (left panel), RSEM (middle panel), and our model fittings (Hebenstreit.a/b, model E; Wei, model B&D; Vahedi, model A). See Table 1 for details of the datasets.

priming and SMART protocols if read numbers are assumed to scale linearly versus length. This is due to the small fraction of a long mRNA that becomes double-stranded cDNA and also implies under-exploitation of the starting material, thus forgoing a potentially higher sensitivity with current scRNA-seq protocols. Inclusion of spike-in probes that are longer than the current standard (< 2 kb) might be valuable for RNA-seq experiments in general to allow for better monitoring of this effect. This might require a different probe production technique from the commonly employed in vitro transcription, which becomes ineffective for long templates.

STAR★METHODS

Detailed methods are provided in the online version of this paper and include the following:

- KEY RESOURCES TABLE
- CONTACT FOR REAGENT AND RESOURCE SHARING
- EXPERIMENTAL MODEL AND SUBJECT DETAILS
    - Mice
- METHOD DETAILS
    - RNA isolation and preparation
    - SMART-seq, SMART-seq2
    - Quartz-seq
    - qPCR & RNA-seq length correlation analysis
    - Data processing
    - Parameter estimation
    - Note - previous approaches to correct coverage bias
    - Models
- QUANTIFICATION AND STATISTICAL ANALYSIS
- DATA AND SOFTWARE AVAILABILITY
    - Data Resources

SUPPLEMENTAL INFORMATION

Supplemental Information includes eight figures and two tables and can be found with this article online at http://dx.doi.org/10.1016/j.cels.2016.10.012.

AUTHOR CONTRIBUTIONS

Conceptualization, D.H.; Methodology, D.H. and V.S.; Software V.S. and D.H.; Formal analysis, D.H. and V.S.; Investigation, N.A. and M.W.; Writing – Original Draft, D.H.; Writing – Review & Editing, D.H., V.S., N.A., and M.W.; Funding Acquisition, D.H.; Supervision, D.H. and V.S.

ACKNOWLEDGMENTS

We would like to thank Mistianne Feeney, Malika Saint, Francois Bertaux, and Sam Marguerat for critically reading the manuscript. The work was supported by Royal Society research grant RG130085, BBSRC research grant BB/L006340/1, and BBSRC research grant BB/M017982/1.

Received: April 11, 2016
Revised: July 28, 2016
Accepted: October 13, 2016
Published: November 10, 2016

REFERENCES

Adiconis, X., Borges-Rivera, D., Satija, R., DeLuca, D.S., Busby, M.A., Berlin, A.M., Sivachenko, A., Thompson, D.A., Wysoker, A., Fennell, T., et al. (2013). Comparative analysis of RNA sequencing methods for degraded or low-input samples. Nat. Methods *10*, 623–629.

Bambara, R.A., Fay, P.J., and Mallaber, L.M. (1995). Methods of analyzing processivity. Methods Enzymol. *262*, 270–280.

Bhargava, V., Head, S.R., Ordoukhanian, P., Mercola, M., and Subramaniam, S. (2014). Technical variations in low-input RNA-seq methodologies. Sci. Rep. *4*, 3678.

Bibillo, A., and Eickbush, T.H. (2002). High processivity of the reverse transcriptase from a non-long terminal repeat retrotransposon. J. Biol. Chem. *277*, 34836–34845.

Bohnert, R., and Rätsch, G. (2010). rQuant.web: A tool for RNA-Seq-based transcript quantitation. Nucleic Acids Res. *38*, W348–W351.

CSHL (2005). Synthesis of complementary DNA. Nat. Methods *2*, 151–152.

Deng, Q., Ramsköld, D., Reinius, B., and Sandberg, R. (2014). Single-cell RNA-seq reveals dynamic, random monoallelic gene expression in mammalian cells. Science *343*, 193–196.

Gansauge, M.T., and Meyer, M. (2013). Single-stranded DNA library preparation for the sequencing of ancient or damaged DNA. Nat. Protoc. *8*, 737–748.

Griebel, T., Zacher, B., Ribeca, P., Raineri, E., Lacroix, V., Guigó, R., and Sammeth, M. (2012). Modelling and simulating generic RNA-Seq experiments with the flux simulator. Nucleic Acids Res. *40*, 10073–10083.

Grün, D., Kester, L., and van Oudenaarden, A. (2014). Validation of noise models for single-cell transcriptomics. Nat. Methods *11*, 637–640.

Haario, H., Saksman, E., and Tamminen, J. (2001). An adaptive metropolis algorithm. Bernoulli *7*, 223–242.

Hansen, K.D., Brenner, S.E., and Dudoit, S. (2010). Biases in Illumina transcriptome sequencing caused by random hexamer priming. Nucleic Acids Res. *38*, e131.

Hashimshony, T., Wagner, F., Sher, N., and Yanai, I. (2012). CEL-Seq: Single-cell RNA-Seq by multiplexed linear amplification. Cell Rep. *2*, 666–673.

Hashimshony, T., Senderovich, N., Avital, G., Klochendler, A., de Leeuw, Y., Anavy, L., Gennert, D., Li, S., Livak, K.J., Rozenblatt-Rosen, O., et al. (2016). CEL-Seq2: Sensitive highly-multiplexed single-cell RNA-Seq. Genome Biol. *17*, 77.

Hebenstreit, D. (2012). Methods, challenges and potentials of single cell RNA-seq. Biology (Basel) *1*, 658–667.

Hebenstreit, D., Fang, M., Gu, M., Charoensawan, V., van Oudenaarden, A., and Teichmann, S.A. (2011). RNA sequencing reveals two major classes of gene expression levels in metazoan cells. Mol. Syst. Biol. *7*, 497.

Houseley, J., and Tollervey, D. (2009). The many pathways of RNA degradation. Cell *136*, 763–776.

Howard, B.E., and Heber, S. (2010). Towards reliable isoform quantification using RNA-SEQ data. BMC Bioinformatics *11* (*Suppl 3*), S6.

Hu, Y., Liu, Y., Mao, X., Jia, C., Ferguson, J.F., Xue, C., Reilly, M.P., Li, H., and Li, M. (2014). PennSeq: Accurate isoform-specific gene expression quantification in RNA-Seq by modeling non-uniform read distribution. Nucleic Acids Res. *42*, e20.

Huang, Y., Hu, Y., Jones, C.D., MacLeod, J.N., Chiang, D.Y., Liu, Y., Prins, J.F., and Liu, J. (2013). A robust method for transcript quantification with RNA-seq data. J. Comput. Biol. *20*, 167–187.

Islam, S., Kjällquist, U., Moliner, A., Zajac, P., Fan, J.B., Lönnerberg, P., and Linnarsson, S. (2012). Highly multiplexed and strand-specific single-cell RNA 5′ end sequencing. Nat. Protoc. *7*, 813–828.

Islam, S., Zeisel, A., Joost, S., La Manno, G., Zajac, P., Kasper, M., Lönnerberg, P., and Linnarsson, S. (2014). Quantitative single-cell RNA-seq with unique molecular identifiers. Nat. Methods *11*, 163–166.

Jiang, L., Schlesinger, F., Davis, C.A., Zhang, Y., Li, R., Salit, M., Gingeras, T.R., and Oliver, B. (2011). Synthetic spike-in standards for RNA-seq experiments. Genome Res. *21*, 1543–1551.

Joseph, S., and David, W.R. (2001). Molecular Cloning: A Laboratory Manual (Cold Spring Harbor Laboratory Press).

Kim, J.K., and Marioni, J.C. (2013). Inferring the kinetics of stochastic gene expression from single-cell RNA-sequencing data. Genome Biol. *14*, R7.

Langmead, B., Trapnell, C., Pop, M., and Salzberg, S.L. (2009). Ultrafast and memory-efficient alignment of short DNA sequences to the human genome. Genome Biol. *10*, R25.

Li, B., and Dewey, C.N. (2011). RSEM: Accurate transcript quantification from RNA-Seq data with or without a reference genome. BMC Bioinformatics *12*, 323.

Li, W., and Jiang, T. (2012). Transcriptome assembly and isoform expression level estimation from biased RNA-Seq reads. Bioinformatics *28*, 2914–2921.

Li, B., Ruotti, V., Stewart, R.M., Thomson, J.A., and Dewey, C.N. (2010a). RNA-seq gene expression estimation with read mapping uncertainty. Bioinformatics *26*, 493–500.

Li, J., Jiang, H., and Wong, W.H. (2010b). Modeling non-uniformity in short-read rates in RNA-Seq data. Genome Biol. *11*, R50.

Macosko, E.Z., Basu, A., Satija, R., Nemesh, J., Shekhar, K., Goldman, M., Tirosh, I., Bialas, A.R., Kamitaki, N., Martersteck, E.M., et al. (2015). Highly parallel genome-wide expression profiling of individual cells using nanoliter droplets. Cell *161*, 1202–1214.

Mahata, B., Zhang, X., Kolodziejczyk, A.A., Proserpio, V., Haim-Vilmovsky, L., Taylor, A.E., Hebenstreit, D., Dingler, F.A., Moignard, V., Göttgens, B., et al. (2014). Single-cell RNA sequencing reveals T helper cells synthesizing steroids de novo to contribute to immune homeostasis. Cell Rep. *7*, 1130–1142.

Mortazavi, A., Williams, B.A., McCue, K., Schaeffer, L., and Wold, B. (2008). Mapping and quantifying mammalian transcriptomes by RNA-Seq. Nat. Methods *5*, 621–628.

Nakamura, T., Yabuta, Y., Okamoto, I., Aramaki, S., Yokobayashi, S., Kurimoto, K., Sekiguchi, K., Nakagawa, M., Yamamoto, T., and Saitou, M. (2015). SC3-seq: A method for highly parallel and quantitative measurement of single-cell gene expression. Nucleic Acids Res. *43*, e60.

Ozsolak, F., and Milos, P.M. (2011). Single-molecule direct RNA sequencing without cDNA synthesis. Wiley Interdiscip. Rev. RNA *2*, 565–570.

Picelli, S., Björklund, A.K., Faridani, O.R., Sagasser, S., Winberg, G., and Sandberg, R. (2013). Smart-seq2 for sensitive full-length transcriptome profiling in single cells. Nat. Methods *10*, 1096–1098.

Quail, M.A., Kozarewa, I., Smith, F., Scally, A., Stephens, P.J., Durbin, R., Swerdlow, H., and Turner, D.J. (2008). A large genome center's improvements to the Illumina sequencing system. Nat. Methods *5*, 1005–1010.

Ramsköld, D., Luo, S., Wang, Y.C., Li, R., Deng, Q., Faridani, O.R., Daniels, G.A., Khrebtukova, I., Loring, J.F., Laurent, L.C., et al. (2012). Full-length mRNA-Seq from single-cell levels of RNA and individual circulating tumor cells. Nat. Biotechnol. *30*, 777–782.

Ritz, C., and Spiess, A.-N. (2008). qpcR: An R package for sigmoidal model selection in quantitative real-time polymerase chain reaction analysis. Bioinformatics *24*, 1549–1551.

Roberts, A., Trapnell, C., Donaghey, J., Rinn, J.L., and Pachter, L. (2011). Improving RNA-Seq expression estimates by correcting for fragment bias. Genome Biol. *12*, R22.

Sasagawa, Y., Nikaido, I., Hayashi, T., Danno, H., Uno, K.D., Imai, T., and Ueda, H.R. (2013). Quartz-Seq: A highly reproducible and sensitive single-cell RNA sequencing method, reveals non-genetic gene-expression heterogeneity. Genome Biol. *14*, R31.

Shalek, A.K., Satija, R., Adiconis, X., Gertner, R.S., Gaublomme, J.T., Raychowdhury, R., Schwartz, S., Yosef, N., Malboeuf, C., Lu, D., et al. (2013). Single-cell transcriptomics reveals bimodality in expression and splicing in immune cells. Nature *498*, 236–240.

Shapiro, E., Biezuner, T., and Linnarsson, S. (2013). Single-cell sequencing-based technologies will revolutionize whole-organism science. Nat. Rev. Genet. *14*, 618–630.

Stegle, O., Teichmann, S.A., and Marioni, J.C. (2015). Computational and analytical challenges in single-cell transcriptomics. Nat. Rev. Genet. *16*, 133–145.

Tang, F., Barbacioru, C., Wang, Y., Nordman, E., Lee, C., Xu, N., Wang, X., Bodeau, J., Tuch, B.B., Siddiqui, A., et al. (2009). mRNA-Seq whole-transcriptome analysis of a single cell. Nat. Methods *6*, 377–382.

Tang, F., Lao, K., and Surani, M.A. (2011). Development and applications of single-cell transcriptome analysis. Nat. Methods *8* (4, Suppl), S6–S11.

Tang, D.T., Plessy, C., Salimullah, M., Suzuki, A.M., Calligaris, R., Gustincich, S., and Carninci, P. (2013). Suppression of artifacts and barcode bias in high-throughput transcriptome analyses utilizing template switching. Nucleic Acids Res. *41*, e44.

Trapnell, C., Roberts, A., Goff, L., Pertea, G., Kim, D., Kelley, D.R., Pimentel, H., Salzberg, S.L., Rinn, J.L., and Pachter, L. (2012). Differential gene and transcript expression analysis of RNA-seq experiments with TopHat and Cufflinks. Nat. Protoc. *7*, 562–578.

Tuerk, A., Wiktorin, G., and Güler, S. (2014). Mixture models reveal multiple positional bias types in RNA-Seq data and lead to accurate transcript concentration estimates. bioRxiv. Published online November 24, 2014. http://dx.doi.org/10.1101/011767.

Vahedi, G., Takahashi, H., Nakayamada, S., Sun, H.W., Sartorelli, V., Kanno, Y., and O'Shea, J.J. (2012). STATs shape the active enhancer landscape of T cell populations. Cell *151*, 981–993.

Von Hippel, P.H., Fairfield, F.R., and Dolejsi, M.K. (1994). On the processivity of polymerases. Ann. N Y Acad. Sci. *726*, 118–131.

Wan, L., Yan, X., Chen, T., and Sun, F. (2012). Modeling RNA degradation for RNA-Seq with applications. Biostatistics *13*, 734–747.

Wang, Z., Gerstein, M., and Snyder, M. (2009). RNA-Seq: A revolutionary tool for transcriptomics. Nat. Rev. Genet. *10*, 57–63.

Wei, G., Abraham, B.J., Yagi, R., Jothi, R., Cui, K., Sharma, S., Narlikar, L., Northrup, D.L., Tang, Q., Paul, W.E., et al. (2011). Genome-wide analyses of transcription factor GATA3-mediated gene regulation in distinct T cell types. Immunity *35*, 299–311.

Wu, Z., Wang, X., and Zhang, X. (2011). Using non-uniform read distribution models to improve isoform expression inference in RNA-Seq. Bioinformatics *27*, 502–508.

Zheng, W., Chung, L.M., and Zhao, H. (2011). Bias detection and correction in RNA-Sequencing data. BMC Bioinformatics *12*, 290.

Zhu, Y.Y., Machleder, E.M., Chenchik, A., Li, R., and Siebert, P.D. (2001). Reverse transcriptase template switching: A SMART approach for full-length cDNA library construction. Biotechniques *30*, 892–897.

STAR★METHODS

KEY RESOURCES TABLE

REAGENT or RESOURCE	SOURCE	IDENTIFIER
Antibodies		
Mouse FITC CD5+	eBioscience	11-0051-81; RRID: AB_464907
Chemicals, Peptides, and Recombinant Proteins		
Smart MMLV Reverse Transcriptase	Clontech	Cat#639524
Kappa Taq	KappaBiosystems	Cat#07958447001
SMARTer cDNA Synthesis Kit	Clontech	Cat#634925
RNase Inhibitor, Murine	New England Biolabs	Cat#M0314
Control Mouse Liver Total RNA	Clontech	Cat#636603
Sensimix SYBR No-Rox	BioLine	Cat#QT650-05
RNase H	ThermoFisher	Cat#EN0201
SuperScript II	ThermoFisher	Cat#18064014
dNTPs (Individual)	New England Biolabs	Part#N0446S
Lympholyte-M Cell Separation Media	Cedarlane labs	Code#CL5030
Exonuclease I	New England Biolabs	Part#M0293
Deoxynucleotide (dNTP) Solution Set	New England Biolabs	Part#N0446S
Terminal deoxynucleotidyl transferase (TdT)	Promega	Cat#M1871
NP-40	ThermoFisher	Cat#28324
Agencourt Ampure XP Beads	Beckman Coulter	Part#A63881
polyA Spin mRNA Isolation Kit	New England Biolabs	Cat#S1560S
TRIzol Reagent	Thermofisher	Cat#15596018
NEB next qPCR quantification kit	New England Biolabs	Cat#E7630L
Critical Commercial Assays		
Nextera XT DNA Library Preparation kit	Illumina	Cat#FC-131-1024
Nextera XT index kit	Illumina	Cat#FC-131-1001
Miseq Reagent kit v3 (150 cycle)	Illumina	Cat#MS-102-3001
Qubit Fluorometric Quantitation	Thermofisher	Cat#Q32866
Agilent 2100 high sensitivity kit	Agilent Technologies	G2939AA
Deposited Data		
Data Files for RNA sequencing	This Paper	GEO: GSE84785
For previously published datasets used in this study see Table 1	Various	N/A
Experimental Models: Organisms/Strains		
Mouse B6CBF1 wildtype	Rodent Facility, University of Warwick	N/A
Sequence-Based Reagents		
For qPCR primers see Table S2	This Paper	N/A
Smart-seq 2 Template Switching Oligonucleotide AAGCAGTGGTATCAA CGCAGAGTACrGrG+G	Picelli et al., 2013	N/A
Quartz-seq RT primer (WTA) TATAGAATT CGCGGCCGCTCGCGATAATACGACTC ACTATAGGGCGTTTTTTTTTTTTTTTTTTT TTTTTT	Sasagawa et al., 2013	N/A
Quartz-seq Tagging primer TATAGAATTC GCGGCCGCTCGCGATTTTTTTTTTTTTTTT TTTTTTTTT	Sasagawa et al., 2013	N/A

(*Continued on next page*)

Continued		
REAGENT or RESOURCE	SOURCE	IDENTIFIER
Quartz-seq suppression PCR primer /5AmMC6/GTATAGAATTCGC GGCCGCTCGCGAT	Sasagawa et al., 2013	N/A
ERCC Spike-in mix	Ambion (Thermofisher)	Cat#4456740
Software and Algorithms		
Mathematica (v10.4)	Wolfram Research, Inc.	http://www.wolfram.com/education/
Bowtie (v1.0.0)	Langmead et al., 2009	http://bowtie-bio.sourceforge.net/index.shtml
MATLAB MCMC toolbox	Haario et al., 2001	http://helios.fmi.fi/~lainema/mcmc
MATLAB and Statistics Toolbox	The MathWorks, Inc.	http://www.mathworks.com/includes_content/domainRedirect/domainRedirect.html?uri=http%3A%2F%2Fuk.mathworks.com%2F
Cufflinks (v2.2.1)	Trapnell et al., 2012	http://cole-trapnell-lab.github.io/cufflinks/
SRA Toolkit	NCBI	http://trace.ncbi.nlm.nih.gov/Traces/sra/sra.cgi
RSEM	Li and Dewey, 2011	http://deweylab.biostat.wisc.edu/rsem/
qpcR R library	Ritz and Spiess, 2008	https://cran.r-project.org/web/packages/qpcR/index.html

CONTACT FOR REAGENT AND RESOURCE SHARING

Further information and requests for reagents may be directed to Lead Contact Daniel Hebenstreit (D.Hebenstreit@warwick.ac.uk).

EXPERIMENTAL MODEL AND SUBJECT DETAILS

Mice

All projects involving animals, including studies not subject to home office licensing are scrutinised and approved by AWERB, established with Home Office guidance and RSPCA/LASA guiding principles on good practice for local ethical review processes. The AWERB ensures that in all cases staff and students are trained and appropriately experienced and that the potential benefits of the research outweigh the effects on the animals concerned while being committed to the promotion of 3Rs (reduction, refinement and replacement). Wild-type B6CBF1 mice were used for splenocyte isolation from spleen. Total mouse RNA used in some experiments was purchased from Clontech.

METHOD DETAILS

RNA isolation and preparation

Starting RNA was either “mouse liver control RNA” (Clontech), or was prepared from murine lymphocytes that were isolated from a B6CBF1 mouse spleen by homogenization through a cell strainer in DMEM-10 media, followed by centrifugation through Lympholyte Ficoll (Cedarlane). For single cell preparations, lymphocytes were additionally stained with anti-CD5 FITC antibody and sorted using a FACS aria fusion (BD Bioscience) into lysis buffer immediately before first-strand synthesis (see below). Otherwise, total RNA was extracted from the lymphocyte suspension using 1 mL Trizol (Ambion) and isolated with 500 μL chloroform before being ethanol precipitated. This was followed by poly-A purification for a selection of samples (Table S1) using the polyA Spin mRNA isolation kit (NEB) following the manufacturer’s instructions. ERCC spike-in probes were added to a subset of samples in the following way. 0.2 μL of a 1:10 dilution of ERCC spike-ins (Ambion) were added to 1 μL of 1μg/μL total RNA, before dilution to 100 pg/μL for low input samples, while 1 μl of a 1:10 dilution were included with the samples containing 100 ng of poly-A+ RNA (Table S1). The RNA was then divided into two equal samples for the different first-strand incubation temperatures.

SMART-seq, SMART-seq2

First-strand synthesis was performed in two separate reactions using the SMART cDNA synthesis kit (Takara Clontech), with different temperatures either 25°C or 42°C but otherwise following the manufacturers’ instructions. In brief, 1 μg total RNA or 100 ng poly-A+ RNA from murine lymphocytes or liver in 3.5 μL was mixed with 1 μL of anchored 12 μM Oligo(dT) (3′ SMART CDS Primer II A) and

denatured at 72°C for 3 min in a thermocycler with a heated lid, before the temperature was dropped to the desired first-strand synthesis temperature. The following were then added for a 60 min incubation: 1 μL 10 mM dNTP mix, 1 μL MMLV SMARTscribe reverse transcriptase (Takara Clontech), 0.25 μL RNase inhibitor, 2 μL 5X smart scribe first-strand buffer, 0.25 μL 100 mM DTT, and 1 μL of 12 μM template switching oligo (SMARTer II A Oligonucleotide).

SMART-seq2 was carried out as described in Picelli et al. (Picelli et al., 2013), with either isolated, lysed cells or 10 pg of total input RNA. In brief, a single lysed CD5+ cell or 10 pg of total RNA (0.1 μL of 100 pg/μL RNA) was mixed with 0.3 μL of anchored 12 μM Oligo(dT) (3′ SMART CDS Primer II A) and 0.3 μL of 10 mM dNTPs before being denatured at 72°C as in SMART-seq "1" as above. Upon reaching the desired first-strand synthesis temperature, 0.5 ul 5X first-strand buffer, 0.5 μL betaine, 0.003 μl MgCl2, 0.06 μl DTT (100 mM stock), 0.25 μl LNA template switching 5′ Oligo, 0.06 μl RNase inhibitor, and 0.25 μl reverse transcriptase (100 u/μL) were added. First-strand synthesis was then carried out at either 25°C or 42°C including the SMART-seq2 temperature cycling as in the below table where xx is the first-strand synthesis temperature.

Single cell Smart-seq2 first strand where xx is the chosen first-strand synthesis temperature of 25 or 42

Cycle	Temperature (°C)	Time
1	xx	90 min
2 to 11	50	2 min
2 to 11	xx	2 min
12	xx	15 min
13	4	Infinite hold

After first-strand synthesis, each sample was again divided into two equal samples for the different second-strand incubation temperatures. Second-strand synthesis was performed with TAQ polymerase (KAPPA) in "Buffer A" tris-ammonium sulfate based buffer with 1.5 mM $MgCl_2$ and in the presence of 200 μM dNTPs with 1 μL of 12 μM 5′ PCR primer ('5-AAGCAGTGGTATCAACGCAGAGT-3′) to prime the second-strand synthesis. Samples were incubated for 20 min at either 72°C or 42°C, followed by PCR for a subset of the SMART-seq samples. PCR was included for all SMART-seq2 samples as follows where xx is the second-strand synthesis temperature:

SMART-seq (with PCR) and Smart-seq2 PCR conditions where xx is the chosen second-strand synthesis temperature of 42 or 72

Cycle	Temperature (°C)	Time
1	98	3 min
2 to 19	98	20 s
2 to 19	68	15 s
2 to 19	xx	6 min
20	xx	5 min
21	4	Infinite hold

Qubit analysis ensured that 1 ng of dual stranded input cDNA for the "tagmentation" reaction using Nextera XT (Illumina) carried out following the manufacturer's instructions including a 12 cycle PCR, at which point molecular barcodes were added identifying the reverse transcription conditions. Ampure XP beads were used to purify the reaction products before quantitation and pooling.

Agilent 2100 high sensitivity kit was used to determine the average fragment size, while quantification was done with a Qubit high sensitivity kit, allowing the libraries to be diluted to a final concentration of 4 nM and pooled, this was then confirmed using the NEB next qPCR quantification kit (NEB). The reactions were then denatured with 0.1 N NaOH and 20 pmol sequenced on Illumina MiSeq using reagents kits v3 in a 75 bp paired-end run. The data were deposited at GEO (http://www.ncbi.nlm.nih.gov/geo/, accession number GEO: GSE84785).

Quartz-seq

Quartz-seq was carried out on 100 ng poly-A purified RNA as previously described (Sasagawa et al., 2013) with minor modifications. In brief, 100 ng of poly-A purified RNA in 3.5 μL was mixed with 1 μL of 10 μM RT Primer (TATAGAATTCGCGGCCGCTCGCGATAA TACGACTCACTATAGGGCG$[T]_{24}$) and denatured at 70°C for 90 s in a thermocycler with a heated lid. The following was then added

to each sample at 4°C: 1 µL10 mM dNTP mix, 1 µL MMLV SMARTscribe reverse transcriptase (Takara Clontech), 0.25 µL RNase inhibitor, 2 µL 5X smart scribe first-strand buffer, 0.25 µL 100 mM DTT and 1 µL nuclease free water. First-strand synthesis was then carried out in a thermocycler at either 25°C or 42°C (xx) as below:

Quartz-seq first-strand where xx is the chosen first-strand synthesis temperature of 25 or 42

Cycle	Temperature (°C)	Time
1	4	To begin
2	35	5 min
3	xx	20 min
4	70	10 min
5	4	Infinite hold

Following first-strand synthesis, the primers were removed using Exonuclease I digestion in Kappa PCR “Buffer A” tris-ammonium sulfate based buffer with 1.5 mM $MgCl_2$. Poly(A) tailing of the single stranded cDNA was then carried out using TdT in the presence of 0.15 mM dATP and Rnase H for 50 s. Second-strand synthesis was carried out for 20 min at either 72°C or 42°C using Kappa Taq as previously. PCR enrichment was carried out using TAQ polymerase (KAPPA) in “Buffer A” tris-ammonium sulfate based buffer with 1.5 mM MgCl2 and in the presence of 200 µM dNTPs with 1 µL of 10 µM PCR primer (NH_2)-GTATAGAATTCGCGGCCGCTCGCGAT, with the following PCR program:

Quartz-seq enrichment PCR where xx is the chosen second-strand synthesis temperature of 42 or 72

Cycle	Temperature (°C)	Time
1	68	To begin
2-18	98	10 s
2-18	65	15 s
2-18	xx	5 min
19	xx	5 min
20	4	Infinite hold

qPCR & RNA-seq length correlation analysis

Two poly-A+ RNA samples corresponding to Smartseq.noPCR.a.ng and Smartseq.noPCR.d.ng (Table S1) were subjected to first-strand synthesis as outlined previously, with the exception that the template switching oligo (TSO) was not included. SensiMix SYBR No-ROX kit (Bioline) was used for qPCR following the manufacturer’s protocol, with the exception of a reduced volume to 10 µl. PCR primers were designed to be located close to the 3′ ends of twelve transcripts covering a range of lengths from ~2 to ~22 kb (Table S2). We designed multiple alternative reverse primers for some genes to test precision of the qPCR (Table S2). These gave very similar results which were averaged for analysis. The reactions were carried out on a QIAGEN Rotorgene-Q 5-plex model, running software v2.1.0, using conditions as shown below:

qPCR conditions

Cycle	Temperature (°C)	Time
1	95	10 min
2-45	95	15 s
2-45	60	15 s
2-45	72	15 s

The transcripts’ expression levels were calculated as 2^{-Ct}, where *Ct* was calculated with the *qpcR R* library, and were z-transformed separately for the two RNA samples. The pooled expression levels were plotted against the corresponding transcript lengths and a linear model was fitted with the *R* function *lm*().

In parallel, we calculated expression levels for the RNA-seq samples corresponding to the same RNA starting preparations (Smartseq.noPCR.a.ng and Smartseq.noPCR.d.ng; all temperature variations were used; Table S1). We analyzed the samples with CuffLinks, RSEM, or fitted our Model D as described below. We averaged expression levels for all temperatures variations of the same starting RNA, and processed the data further in the same way as the qPCR data.

Data processing

Datasets (see Table 1 for accession codes) were downloaded from GEO (http://www.ncbi.nlm.nih.gov/geo/) or ENCODE (https://www.encodeproject.org/). SRA format files were converted to FASTQ format files using the fastq-dump program from the SRA Toolkit (https://trace.ncbi.nlm.nih.gov/Traces/sra/sra.cgi?view=toolkit_doc). We downloaded RefSeq gene annotations for the mouse genome (mm10) from UCSC Genome Browser (https://genome.ucsc.edu/). We then used custom Perl scripts to remove all entries that overlapped each other or corresponded to multiple isoforms of a gene. The mRNA sequences for the remaining entries were downloaded from UCSC Genome Browser and were clipped by any 3′ stretches of poly-A. In addition, we downloaded a FASTA file for the ERCC-mix1 spike-in controls from https://www.encodeproject.org/datasets/ENCSR156CIL/. Both sets of sequences were used to generate indices for Bowtie 1.0.0 (Langmead et al., 2009). All datasets were then mapped to the appropriate indices with Bowtie, using option '-m 1'. The starting positions of reads were extracted from the mapping output files and were collected as lists for each transcript. Names, lengths, and read position lists of each transcript were saved into files, which were used as input for the parameter estimations.

Datasets produced in this study were processed as described above, with 'NR_' transcripts removed from the annotation file for most analyses due to an outlying ribosomal RNA that was present also in all poly-A+ samples. Unique read mappings were ~20 to 50% for poly-A+ RNA and ~2 to 5% for total RNA. Please note that our parameter estimations do not require large read numbers; we therefore aimed for ~0.1M to ~1M reads per individual sample (Table S1).

To calculate FPKM using the bias correction approach by Roberts et al. (Roberts et al., 2011), we downloaded and ran CuffLinks 2.2.1 (http://cole-trapnell-lab.github.io/cufflinks/cuffdiff/) (Trapnell et al., 2012). We supplied it with a GTF and a FASTA file prepared from our transcript sequences, using options *-G* and *-b*, respectively.

To calculate FPKM using the RSEM bias correction approach (Li and Dewey, 2011), we downloaded RSEM 1.2.22 software (http://deweylab.biostat.wisc.edu/rsem/) and used the function *rsem-prepare-reference* to produce an RSEM index for our transcript sequences. We then ran *rsem-calculate-expression* using options *–sam*, *–estimate-rspd* and *–no-bam-output,* and in addition *–paired-end* for the Mahata dataset.

FPKM for spike-in probes in newly generated RNA-seq samples were calculated using the standard formula (10^9 × reads × probe-length^{-1} × total-reads^{-1}), where total reads were all reads mapping to the spike-in probes.

The coverage plots were generated in *Mathematica* 10 using the function *ArrayPlot* after calculating the densities of read starting positions in 20 equally sized bins along the transcript lengths. The *ColorFunction* in *ArrayPlot* was set to *ColorData*["*SunsetColors*"][1 - 10 #] &. The same binned data were used to compare coverage uniformity in the following way; RNA-seq samples prepared at standard temperatures and the corresponding low-temperature samples were compared in terms of the numbers of detected genes. All genes of the sample with the lower number were used for further processing. A random sample of the same number of genes was selected from the other sample. This was done in order to process equal numbers of genes for the two samples. We then calculated the statistical entropy for the binned data of all genes in both samples, since entropy becomes maximal for uniform distributions. We then calculated the medians of these distributions and their ratio regarding high- and low-temperature samples.

Parameter estimation

We used a Bayesian Markov Chain Monte Carlo (MCMC) Framework to fit the RNA-seq data and infer parameters of each model. The number of reads processed for a transcript was limited to 100, to reduce computational time. We confirmed including more data did not affect our parameter estimates. We further used similar read numbers for different ranges of gene lengths to obtain an unbiased estimate across all transcript lengths. We used likelihood ratios to test the goodness-of-fits of the models. Inference was performed using the MATLAB MCMC toolbox (http://helios.fmi.fi/~lainema/mcmc/). For the parameter perturbations and bias correction analyses (Figures 3 and 4), we fit Model B&D to SMART-seq/SMART-seq2 samples, Model D to all others (including SMART-seq without flanking PCR).

Note - previous approaches to correct coverage bias

Correction of RNA-seq coverage bias is necessary in order to yield correct estimates of the original mRNAs' abundances. Effects such as mRNA secondary structure or usage of random-primers with non-uniform nucleotide frequencies will influence the read distributions within transcripts. However, this type of coverage bias affects individual transcripts in a sequence-specific manner. In contrast, the enzymatic bias that we address affects the representation of transcripts in a systematic and length-dependent way; it can result in different shapes of the coverage distribution at different lengths. It is equally possible that the shape stays the same while the total expected read numbers per transcript vary greatly and/or disproportionately for different lengths. Successful correction of this bias thus requires an understanding of the actual, potentially non-linear, scaling between numbers of sequencing reads mapping to transcripts and their lengths. While several methods to correct RNA-seq coverage bias have been published, they mostly fall short with regards to this last point.

Most methods follow the same overall strategy of re-weighing read densities along transcripts based on functions that describe their deviance from uniform distributions. These functions range from non-parametric empirical to stepwise linear to Gaussian mixture models and others (Bohnert and Rätsch, 2010; Li et al., 2010a; Howard and Heber. 2010; Wu et al., 2011; Hu et al., 2014; Roberts et al., 2011; Li and Jiang, 2012; Huang et al., 2013; Tuerk et al., 2014). The approaches also differ by their resolution (bins, sections, single bases, etc.), by how the functions are learned, and whether/how the functions are allowed to vary with transcript length. However, none of these methods offer mechanistic insights or scale across transcripts as explained above since they are not based on an understanding of the logics underlying library preparation.

An approach that suffers from similar problems but takes into account scaling across transcripts is provided by Zheng et al. (2011). The authors aim to remove length-related bias, which is implicitly based on the assumption that mRNA length and expression levels are independent variables. It is not clear that this would be so as, biologically, restricting expression of long transcripts might be energetically favorable; in line with this, even the (largely systematic bias-free-) RNA-fragmentation protocol displays anti-correlation between length and expression level (Figure S8b, Vahedi dataset). Also, we have observed anti-correlation between gene length and expression level in most datasets after correcting for the length bias using our methodology (Figure S8b).

Noteworthy is also the approach of Wan et al. (2012), who correct FPKM within exons by a factor that is taken to exponentially decrease from the 3′ ends to the center of each exon. This is somewhat similar to our model B (Figure 1D), although their factor is fitted separately for each gene. The authors interpret the exponential decrease as mRNA decay, which appears implausible for a number of reasons; RNA degradation during experimental procedures is improbable to occur from one side only and/or to occur at rates that correlate across datasets (Wan et al., 2012). Biological degradation, on the other hand, is unlikely to lead to a simple exponential decrease; processive, one-sided degradation should theoretically yield a shape corresponding $1-e^{-\delta x}$(δ being the degradation rate), if detectable at all; biological mRNA degradation takes place extremely rapidly, usually leaving no detectable intermediates Houseley and Tollervey (2009). Note also that coverage bias is not generally limited to one side. We show that the bias can shift from 3′ to 5′ end, or be bimodal, or be absent for the same cell type (Th2 cells, compare the datasets Wei, Hebenstreit, Mahata, Vahedi, respectively) depending on the library preparation protocol (Figure S1). Our model predicts and confirms this effect.

It is worth including a brief discussion of the ‘Flux Simulator’ (Griebel et al., 2012) tool here. Although it is not a bias correction method, it aims to computationally simulate the steps of experimental protocols in terms of their influence on the resulting read distributions. It is used for in silico data generation in several studies and is interesting for its consideration of enzymatic reactions during library preparation. The dependencies of first- and second-strand syntheses on priming strategies are recognized. However, parameter estimations for these are not possible and the software makes extensive simplifications; syntheses endpoints are assumed to be uniformly distributed and are limited to a maximum distance of 5 kb. Over-represented ends of (short) strands upon frequent full-length syntheses are not considered either (compare our density functions for synthesis endpoints, which feature delta peaks at the ends; Figure S2b).

Models

Preliminaries

We use conditional probabilities for different events within an experimental protocol to derive an expression for the likelihood of a sequencing read start position. For instance, the probability to initiate reverse transcription/first-strand synthesis might depend on the binding probability of a primer to the mRNA, which in turn might depend on the transcript length, etc. These individual dependencies can be conveniently factored following the chain rule to give the joint probability.

For one particular transcript of length l, let the possible start- and end-points of first- and second-strand cDNA synthesis be denoted by the random variables s_1, e_1, s_2 and e_2 (all $\in \mathbb{R}$), respectively, as illustrated in Figure S2a.

To take account of (i) the opposite direction of first- and second-strand syntheses and of (ii) the fact that synthesis necessarily ends at either end of the transcript, the following semi-continuous conditional probability densities are introduced (Figure S2b):

$$p(e_1 \mid s_1) = \begin{cases} \varphi(s_1 - e_1, \theta_1) + \delta(e_1)[1 - \Phi(s_1, \theta_1)] & \text{if } 0 \le e_1 \le s_1 \le l, \\ 0 & \text{otherwise.} \end{cases}$$

and

$$p(e_2 \mid s_2) = \begin{cases} \varphi(e_2 - s_2, \theta_2) + \delta(e_2 - l)[1 - \Phi(l - s_2, \theta_2)] & \text{if } 0 \le s_2 \le e_2 \le l, \\ 0 & \text{otherwise.} \end{cases}$$

Here, φ gives probability densities for the synthesis lengths of first- and second-strand enzymes (reverse transciptase and DNA polymerase), respectively, and Φ gives the corresponding cumulative distribution functions. $\delta(x)$ represented the Dirac delta function. The distributions are subject to parameters θ_1 and θ_2, and can be assumed to be exponential distributions based on what is known for the processivity of polymerases (Figure S2b; in the case of concatenates of several individual polymerization processes, sums of exponentially distributed variables will result, forming Erlang distributions).

We then have

$$\varphi(x, \theta) = \theta e^{-\theta x}; \quad \Phi(x, \theta) = 1 - e^{-\theta x}.$$

Given the above conditional probabilities, in the following we will use the following relation:

$$p(s_1,e_1,s_2,e_2)=p(e_2\,|\,s_2,s_1)p(s_2\,|\,e_1,s_1)p(e_1\,|\,s_1)p(s_1).$$

If as in the models A to D (Figure 1D), we have $p(s_1)=\delta(s_1-l)$, the dependence of s_2 and e_2 on s_1 is dropped. In many protocols the cDNA is fragmented before sequencing. We assume there is uniform probability of fragmentation along the cDNA and therefore there is also uniform probability of a sequencing start read along the transcript's length. However, we assume that fragmentation efficiency is reduced for positions closer than a distance h from either end, thus resulting in lower sequencing coverage. We calculate the likelihood of a sequencing read on a fragmented end of a cDNA in either direction for a given mRNA as

$$f_{frag}(x)=\begin{cases}\frac{1}{d}P(s_2<x<e_2)+P(s_2+h<x<e_2-h) & \text{for } h<x<l-h,\\ \frac{1}{d}P(s_2<x<e_2) & \text{otherwise.}\end{cases}$$

Coverage inside fragments is thus assumed to be higher by a factor $(d+1)$ than close to ends (we assume $d>0$).

In the following, we derive analytical expressions for the likelihood function for several models of how library preparation of various RNA-seq protocols takes place (Figure 1D in the main text).

Derivations of Models

Symbolic calculations of the derivations below were carried out with *Mathematica* software and were checked manually where feasible.

Derivation of Model A. This model is compatible with idealistic assumptions about SMART-based protocols; first-strand synthesis is primed with oligo(dT) primers and thus starts at the 3′ end of transcripts (i.e., at position l). Only first-strands reaching the 5′ ends of transcripts are primed for second-strand synthesis at position 0. Only second-strands reaching position l are processed for sequencing. Therefore:

$$p(s_1)=\delta(s_1-l)\text{ and }p(s_2\,|\,e_1)=\delta(e_1)\delta(s_2).$$

We have for for $h<x<l-h$

$$\begin{aligned}P_A(s_2+h<x<e_2-h)&=\int_0^l p(s_2+h<x<e_2-h)p(s_1,e_1,s_2,e_2)ds_1de_1ds_2de_2\\ &=\int_0^l ds_1de_1\int_{x+h}^l de_2\delta(e_2-l)\int_0^{x-h}ds_2p(e_2,s_2,e_1,s_1)\\ &=\int_0^l de_1\int_{x+h}^l de_2\delta(e_2-l)\int_0^{x-h}ds_2p(e_2\,|\,s_2)p(s_2\,|\,e_1)p(e_1\,|\,l)\\ &=\int_{x+h}^l de_2\delta(e_2-l)\int_0^{x-h}ds_2p(e_2\,|\,s_2)\delta(s_2)[1-\Phi(l,\theta_1)]\\ &=[1-\Phi(l,\theta_1)]\,[1-\Phi(l,\theta_2)]\\ &=e^{-l(\theta_1+\theta_2)}.\end{aligned}$$

We note that for $h>x$ or $x>l-h$ we have

$$P_A(s_2+h<x<e_2-h)=0.$$

We note that the integrals above are a continuous approximation of the discrete sums over mRNA residues that includes the 3′ and 5′ end of the mRNA. So, the integral limits are from $0-\varepsilon$ to $l+\varepsilon$, where ε is a small positive real number. Throughout, these limits are taken into account when integrating over the Dirac δ functions but for simplicity the ε's are not explicitly included.

Derivation of Model B. This model is compatible with idealistic assumptions about poly-A-tagging protocols; first-strand synthesis is primed with oligo(dT) primers and thus starts at the 3′ end of transcripts (i.e., at position l). Second-strand synthesis starts at the end of first-strands. Only second-strands reaching position l are processed for sequencing. Therefore:

$$p(s_1)=\delta(s_1-l)\text{ and }p(s_2\,|\,e_1)=\delta(s_2-e_1).$$

OPEN ACCESS
CellPress

We have for $h < x < l - h$

$$P_B(s_2 + h < x < e_2 - h) = \int_0^l p(s_2 + h < x < e_2 - h)p(s_1, e_1, s_2, e_2)ds_1 de_1 ds_2 de_2$$

$$= \int_0^l ds_1 de_1 \int_{x+h}^l de_2\, \delta(e_2 - l) \int_0^{x-h} ds_2 p(e_2, s_2, e_1, s_1)$$

$$= \int_0^l de_1 \int_{x+h}^l de_2\, \delta(e_2 - l) \int_0^{x-h} ds_2 p(e_2 \,|\, s_2)p(s_2 \,|\, e_1)p(e_1 \,|\, l)$$

$$= \int_{x+h}^l de_2\, \delta(e_2 - l) \int_0^{x-h} ds_2 p(e_2 \,|\, s_2)p(s_2 \,|\, l)$$

$$= \int_0^{x-h} ds_2\, p(s_2 \,|\, l) \int_{x+h}^l de_2\, \delta(e_2 - l)p(e_2 \,|\, s_2)$$

$$= \int_0^{x-h} ds_2\, p(s_2 \,|\, l)p(l \,|\, s_2)$$

$$= \int_0^{x-h} ds_2\, p(s_2 \,|\, l)[1 - \Phi(l - s_2, \theta_2)]$$

$$= \frac{1}{(\theta_1 + \theta_2)} \left[\theta_1 e^{-2l(\theta_1 + \theta_2) + (\theta_1 + \theta_2)(l - h + x)} + \theta_2 e^{-l(\theta_1 + \theta_2)}\right].$$

We note again that for $h > x$ or $x > l - h$ we have

$$P_B(s_2 + h < x < e_2 - h) = 0.$$

Derivation of Model C. This model assumes that full-length first-strands are selected, while second-strand synthesis may be incomplete; first-strand synthesis is primed with oligo(dT) primers and thus starts at the 3′ end of transcripts (i.e., at position *l*). Only first-strands reaching the 5′ ends of transcripts are primed for second-strand synthesis at position 0. Therefore:

$$p(s_1) = \delta(s_1 - l) \text{ and } p(s_2 \,|\, e_1) = \delta(e_1)\delta(s_2).$$

We have for for $h < x < l - h$

$$P_C(s_2 + h < x < e_2 - h) = \int_0^l p(s_2 + h < x < e_2 - h)p(s_1, e_1, s_2, e_2)ds_1 de_1 ds_2 de_2$$

$$= \int_0^l ds_1 de_1 \int_{x+h}^l de_2 \int_0^{x-h} ds_2\, p(e_2, s_2, e_1, s_1)$$

$$= \int_0^l ds_1 de_1 \int_{x+h}^l de_2 \int_0^{x-h} ds_2\, p(e_2 \,|\, s_2)p(s_2 \,|\, e_1)p(e_1 \,|\, s_1)p(s_1)$$

$$= [1 - \Phi(l, \theta_1)]\, [1 - \Phi(x + h, \theta_2)]$$

$$= e^{-\theta_1 l - \theta_2 (x + h)}.$$

We again note that for $h > x$ or $x > l - h$ we have

$$P_C(s_2 + h < x < e_2 - h) = 0.$$

Derivation of Model D. This model assumes that no selection for full-length syntheses takes place and is compatible with imperfect SMART or poly-A-tagging protocols; first-strand synthesis is primed with oligo(dT) primers and thus starts at the 3′ end of transcripts (i.e., at position *l*). Second-strand synthesis starts at the end of first-strands. Therefore:

$$p(s_1) = \delta(s_1 - l) \text{ and } p(s_2 \,|\, e_1) = \delta(s_2 - e_1).$$

OPEN ACCESS
CellPress

We have for $h < x < l - h$

$$P_D(s_2 + h < x < e_2 - h) = \int_0^l p(s_2 + h < x < e_2 - h) p(s_1, e_1, s_2, e_2) ds_1 de_1 ds_2 de_2$$

$$= \int_0^l ds_1 de_1 \int_{x+h}^l de_2 \int_0^{x-h} ds_2 p(e_2, s_2, e_1, s_1)$$

$$= \int_0^l ds_1 de_1 \int_{x+h}^l de_2 \int_0^{x-h} ds_2 \, p(e_2 \mid s_2) p(s_2 \mid e_1) p(e_1 \mid s_1) p(s_1)$$

$$= \int_0^{x-h} ds_2 \left[p(e_1 = s_2 \mid s_1 = l) \int_{x+h}^l de_2 p(e_2 \mid s_2) \right]$$

$$= \int_0^{x-h} ds_2 [p(e_1 = s_2 \mid s_1 = l)[1 - \Phi(x + h - s_2, \theta_2)]]$$

$$= \int_0^{x-h} \varphi(l - s_2, \theta_1)[1 - \Phi(x + h - s_2, \theta_2)] ds_2$$

$$+ [1 - \Phi(l, \theta_1)] \, [1 - \Phi(x + h, \theta_2)]$$

$$= \frac{\theta_1}{\theta_1 + \theta_2} \left[e^{-\theta_1 (l - x) - \theta_1 h - 2\theta_2 h} - e^{-\theta_1 l - \theta_2 (x + h)} \right] + e^{-\theta_1 l - \theta_2 (x + h)}$$

$$= \frac{1}{\theta_1 + \theta_2} \left[\theta_1 e^{-\theta_1 (l - x) - \theta_1 h - 2\theta_2 h} + \theta_2 e^{-\theta_1 l - \theta_2 (x + h)} \right].$$

We note that for $h > x$ or $x > l - h$ we have

$$P_D(s_2 + h < x < e_2 - h) = 0.$$

Derivation of Model E. This model is based on random-primed first- and second-strand syntheses. To model first-strand priming, we set

$$p(s_1) = \begin{cases} \alpha_1 & \text{if } 0 \le s_1 \le l, \\ 0 & \text{otherwise.} \end{cases}$$

where, l is the length of mRNA as before and α_1 is the probability of primer binding per position. Second-strand priming is usually carried out by (random) RNaseH nicking, so we have similarly

$$p(s_2 \mid e_1, s_1) = \begin{cases} \alpha_2 & \text{if } e_1 \le s_2 \le s_1, \\ 0 & \text{if } s_2 < e_1 \text{or} s_2 > s_1. \end{cases}$$

We assume that multiple priming events on the same first- and/or second-strand are possible. We approximate the effects of this by assuming it will reduce the average syntheses lengths, yielding modified processivity parameters $\theta_{1'}$ and $\theta_{2'}$. We have:

$$p(e_1 \mid s_1) = \begin{cases} \varphi_1(s_1 - e_1, \theta_1') + \delta(e_1)[1 - \Phi_1(s_1, \theta_1')] & \text{if } 0 \le e_1 \le s_1, \\ 0 & \text{otherwise.} \end{cases}$$

and

$$p(e_2 \mid s_2, s_1) = \begin{cases} \varphi_2(e_2 - s_2, \theta_2') + \delta(e_2 - s_1)[1 - \Phi_2(s_1 - s_2, \theta_2')] & \text{if } s_2 \le e_2 \le s_1, \\ 0 & \text{if } s_2 > e_2 \text{ or } e_2 > s_1. \end{cases}$$

As we are assuming synthesis length follows an exponential decay, the new decay lengths can simply be related to the original ones:

$$\theta_1' = \theta_1 + \alpha_1,$$

$$\theta_2' = \theta_2 + \alpha_2.$$

Using the above probabilities, similar to the last sections, we can calculate the probabilities that are required to derive the likelihoods.

We have for for $h<x<l-h$

$$P_E(s_2+h<x<e_2-h)=\int_0^l p(s_2+h<x<e_2-h)p(s_1,e_1,s_2,e_2)ds_1de_1ds_2de_2$$

$$=\int_0^l ds_1de_1 \int_{x+h}^l de_2 \int_0^{x-h} ds_2\, p(e_2,s_2,e_1,s_1)$$

$$=\int_0^l ds_1de_1 \int_{x+h}^l de_2 \int_0^{x-h} ds_2\, p(e_2\,|\,s_2,s_1)p(s_2\,|\,e_1,s_1)p(e_1\,|\,s_1)p(s_1)$$

$$=\alpha_1\alpha_2 \int_{x+h}^l ds_1 \int_0^{x-h} de_1 \int_{e_1}^{x-h} ds_2 \int_{x+h}^{s_1} de_2\left[\varphi(e_2-s_2,\theta_2')\right.$$

$$\left.+\delta(e_2-s_1)\left[1-\Phi(s_1-s_2,\theta_2')\right]\right]\left[\varphi(s_1-e_1,\theta_1')+\delta(e_1)\left[1-\Phi(s_1,\theta_1')\right]\right]$$

$$=\frac{\alpha_1\alpha_2}{\theta_1'(\theta_1'+\theta_2')}e^{-(2h+l+x)\theta_1'-(2h+x)\theta_2'}\left(e^{l\theta_1'}-e^{(h+x)\theta_1'}\right)\left(e^{x(\theta_1'+\theta_2')}-e^{h(\theta_1'+\theta_2')}\right).$$

We again note that for $h>x$ or $x>l-h$ we have

$$P_E(s_2+h<x<e_2-h)=0.$$

Derivation of Mixture model B&D. For this model, we assume that a partial selection of full-length second-strands takes place based on PCR using 3′ flanking primers. This corresponds to a mixture of models B and D:

$$P_{B\&D}(s_2+h<x<e_2-h)=\alpha P_B+(1-\alpha)P_D,\quad 0\le\alpha\le 1.$$

Model summary and correction (normalization) factors

Model A. Fragmentation model / Coverage function

$$f_{frag}(x)=\begin{cases}\left(\frac{1}{d}+1\right)e^{-l(\theta_1+\theta_2)} & \text{for } h<x<l-h,\\ \frac{1}{d}e^{-l(\theta_1+\theta_2)} & \text{otherwise.}\end{cases}$$

Area under coverage function

$$\int_0^l f_{frag}(x)dx=\begin{cases}\frac{l+d(l-2h)}{d}e^{-l(\theta_1+\theta_2)} & \text{for } h<\frac{l}{2},\\ \frac{l}{d}e^{-l(\theta_1+\theta_2)} & \text{otherwise.}\end{cases}$$

Model B. Fragmentation model / Coverage function

$$f_{frag}(x)=\begin{cases}\frac{1}{d(\theta_1+\theta_2)}\left[\theta_1e^{-2l(\theta_1+\theta_2)+(\theta_1+\theta_2)(l+x)}+\theta_2e^{-l(\theta_1+\theta_2)}\right]+\frac{1}{(\theta_1+\theta_2)}\left[\theta_1e^{-2l(\theta_1+\theta_2)+(\theta_1+\theta_2)(l-h+x)}+\theta_2e^{-l(\theta_1+\theta_2)}\right] & \text{for } h<x<l-h,\\ \frac{1}{d(\theta_1+\theta_2)}\left[\theta_1e^{-2l(\theta_1+\theta_2)+(\theta_1+\theta_2)(l+x)}+\theta_2e^{-l(\theta_1+\theta_2)}\right] & \text{otherwise.}\end{cases}$$

Area under coverage function

$$\int_0^l f_{frag}(x)dx=\begin{cases}\frac{1}{d(\theta_1+\theta_2)^2}\left[\theta_1+e^{-l(\theta_1+\theta_2)}\left(l\theta_2^2+l\theta_1\theta_2-\theta_1\right)\right]+\frac{\theta_1\left(e^{-2h(\theta_1+\theta_2)}-e^{-l(\theta_1+\theta_2)}\right)+\theta_2(\theta_1+\theta_2)(l-2h)e^{-l(\theta_1+\theta_2)}}{(\theta_1+\theta_2)^2} & \text{for } h<\frac{l}{2},\\ \frac{1}{d(\theta_1+\theta_2)^2}\left[\theta_1+e^{-l(\theta_1+\theta_2)}\left(l\theta_2^2+l\theta_1\theta_2-\theta_1\right)\right] & \text{otherwise.}\end{cases}$$

Model C. Fragmentation model / Coverage function

$$f_{frag}(x)=\begin{cases}\frac{1}{d}e^{-\theta_1 l-\theta_2 x}+e^{-\theta_1 l-\theta_2(x+h)} & \text{for } h<x<l-h,\\ \frac{1}{d}e^{-\theta_1 l-\theta_2 x} & \text{otherwise.}\end{cases}$$

Area under coverage function

$$\int_0^l f_{frag}(x)dx=\begin{cases}\frac{e^{-l\theta_1}\left(1-e^{-l\theta_2}\right)}{d\theta_2}+\frac{e^{-l\theta_1}\left(e^{-2h\theta_2}-e^{-l\theta_2}\right)}{\theta_2} & \text{for } h<\frac{l}{2},\\ \frac{e^{-l\theta_1}\left(1-e^{-l\theta_2}\right)}{d\theta_2} & \text{otherwise.}\end{cases}$$

Model D. Fragmentation model / Coverage function

$$f_{frag}(x)=\begin{cases}\frac{1}{d(\theta_1+\theta_2)}\left[\theta_1 e^{-\theta_1(l-x)}+\theta_2 e^{-\theta_1 l-\theta_2 x}\right]+\frac{1}{\theta_1+\theta_2}\left[\theta_1 e^{-\theta_1(l-x)-\theta_1 h-2\theta_2 h}+\theta_2 e^{-\theta_1 l-\theta_2(x+h)}\right] & \text{for } h<x<l-h,\\ \frac{1}{d(\theta_1+\theta_2)}\left[\theta_1 e^{-\theta_1(l-x)}+\theta_2 e^{-\theta_1 l-\theta_2 x}\right] & \text{otherwise.}\end{cases}$$

Area under coverage function

$$\int_0^l f_{frag}(x)dx=\begin{cases}\frac{1-e^{-l(\theta_1+\theta_2)}}{d(\theta_1+\theta_2)}+\frac{e^{-2h(\theta_1+\theta_2)}-e^{-l(\theta_1+\theta_2)}}{\theta_1+\theta_2} & \text{for } h<\frac{l}{2},\\ \frac{1-e^{-l(\theta_1+\theta_2)}}{d(\theta_1+\theta_2)} & \text{otherwise.}\end{cases}$$

Model E. Fragmentation model / Coverage function

$$f_{frag}(x)=\begin{cases}\frac{\alpha_1\alpha_2}{d\theta'_1\left(\theta'_1+\theta'_2\right)}\left(1-e^{-x\left(\theta'_1+\theta'_2\right)}-e^{-(l-x)\theta'_1}+e^{-l\theta'_1-x\theta'_2}\right) & \\ +\frac{\alpha_1\alpha_2}{\theta'_1\left(\theta'_1+\theta'_2\right)}\left(e^{-2h\left(\theta'_1+\theta'_2\right)}-e^{-(h+x)\left(\theta'_1+\theta'_2\right)}-e^{-2h\theta'_2-(l+h-x)\theta'_1}+e^{-l\theta'_1-(h+x)\theta'_2}\right) & \text{for } h<x<l-h,\\ \frac{\alpha_1\alpha_2}{d\theta'_1\left(\theta'_1+\theta'_2\right)}\left(1-e^{-x\left(\theta'_1+\theta'_2\right)}-e^{-(l-x)\theta'_1}+e^{-l\theta'_1-x\theta'_2}\right) & \text{otherwise.}\end{cases}$$

Area under coverage function

$$\int_0^l f_{frag}(x)dx=$$

$$\begin{cases}\frac{\alpha_1\alpha_2}{d\theta'_1\left(\theta'_1+\theta'_2\right)}\left[l-\frac{1}{\theta'_1+\theta'_2}-\frac{1}{\theta'_1}-\frac{\theta'_1 e^{-l\left(\theta'_1+\theta'_2\right)}}{\theta'_2\left(\theta'_1+\theta'_2\right)}+\frac{\left(\theta'_1+\theta'_2\right)\quad e^{-l\theta'_1}}{\theta'_1\theta'_2}\right] & \\ +\frac{\alpha_1\alpha_2}{\theta_1^{2'}\theta'_2\left(\theta'_1+\theta'_2\right)^2}\left[e^{-l\theta'_1-2h\theta'_2}\left(\theta'_1+\theta'_2\right)^2-e^{-l\left(\theta'_1+\theta'_2\right)}\theta_1^{2'}+\theta'_1\theta'_2 e^{-2h\left(\theta'_1+\theta'_2\right)}\left(l\theta'_2-2h\theta'_1-2h\theta'_2+l\theta'_1-\theta'_2/\theta'_1-2\right)\right] & \text{for } h<\frac{l}{2},\\ \frac{\alpha_1\alpha_2}{d\theta'_1\left(\theta'_1+\theta'_2\right)}\left[l-\frac{1}{\theta'_1+\theta'_2}-\frac{1}{\theta'_1}-\frac{\theta'_1 e^{-l\left(\theta'_1+\theta'_2\right)}}{\theta'_2\left(\theta'_1+\theta'_2\right)}+\frac{\left(\theta'_1+\theta'_2\right)\quad e^{-l\theta'_1}}{\theta'_1\theta'_2}\right] & \text{otherwise.}\end{cases}$$

Mixture model B&D. Fragmentation model and correction (normalization) factor correspond to the weighted sum as given above in the derivation of the mixture of models B&D.

QUANTIFICATION AND STATISTICAL ANALYSIS

Statistical parameters and statistical significance are reported in the figures and figure legends. Data were in general judged to be statistically significant when $p < 0.05$ using the statistical tests are described in figure legends. Where data were suspected not to be normally distributed, Mann Whitney U (unpaired data) and Wilcoxon signed-rank tests (paired data) were used as appropriate and as

identified in the figure legends; otherwise one-sided t tests were used as indicated in the figure legends. Trendlines are based on straight-line fits calculated with Mathematica or *R* as described in the method text and figure legends where relevant. Correlations in Figure 4D were calculated with Mathematica 10 and are the Pearson product-moment correlation coefficient.

DATA AND SOFTWARE AVAILABILITY

Data Resources

The accession number for the raw and processed data files for the RNA sequencing analysis reported in this paper is NCBI GEO: GSE84785.

Cell Systems, Volume *3*

Supplemental Information

Modeling Enzyme Processivity Reveals that RNA-Seq Libraries Are Biased in Characteristic and Correctable Ways

Nathan Archer, Mark D. Walsh, Vahid Shahrezaei, and Daniel Hebenstreit

SUPPLEMENTAL FIGURES

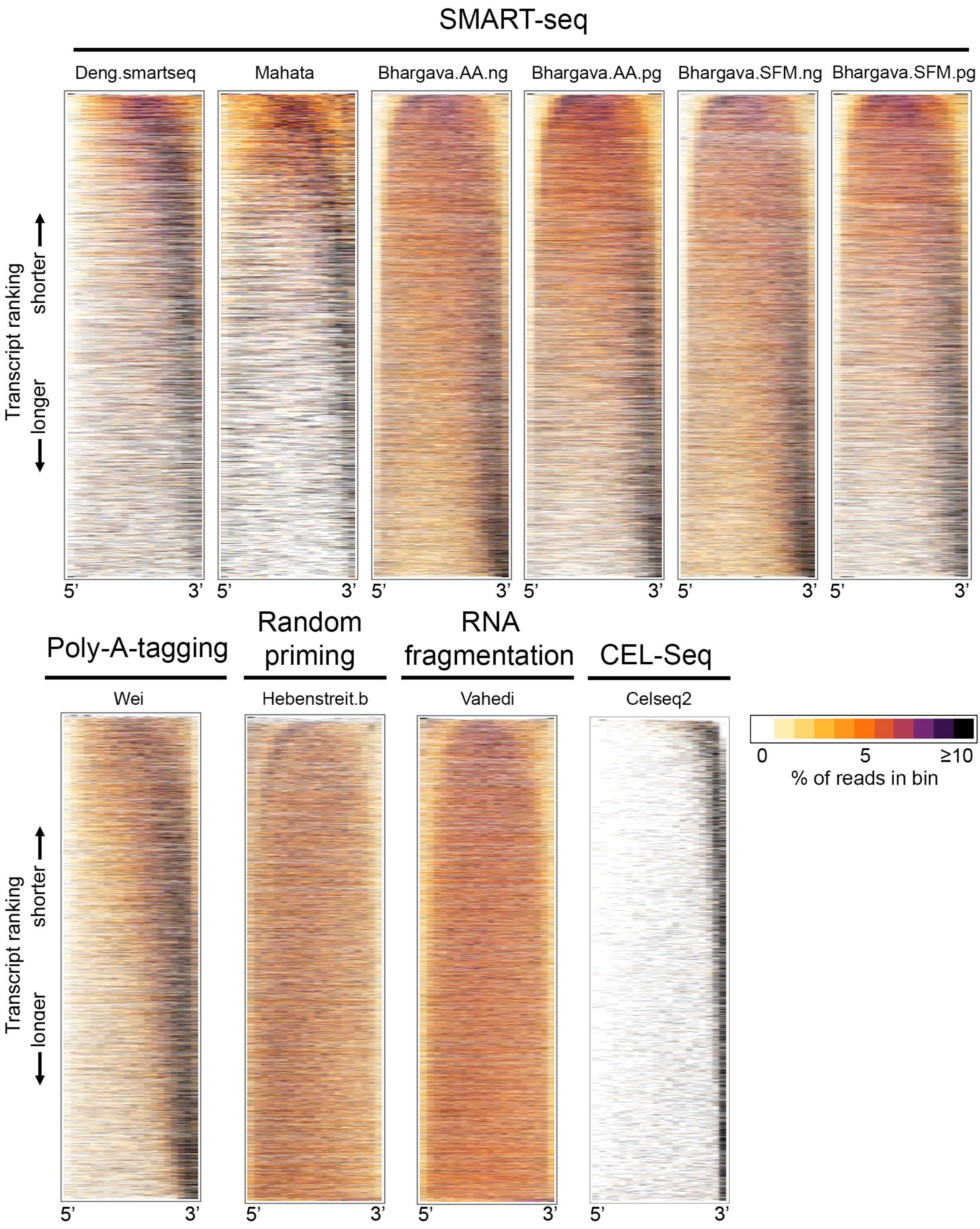

Figure S1. **Related to Figure 1. Related to Table 1**. RNA-seq coverage along transcripts for different datasets as in Figure 1C. Details of the datasets shown are listed in Table 1.

a

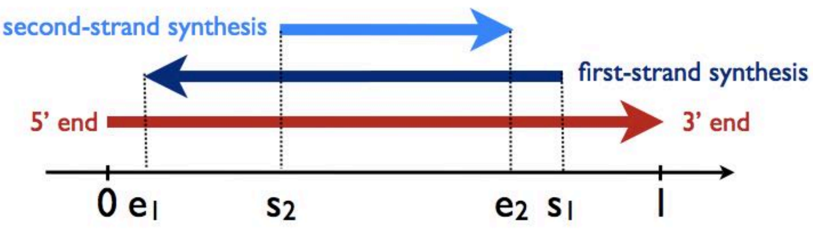

b

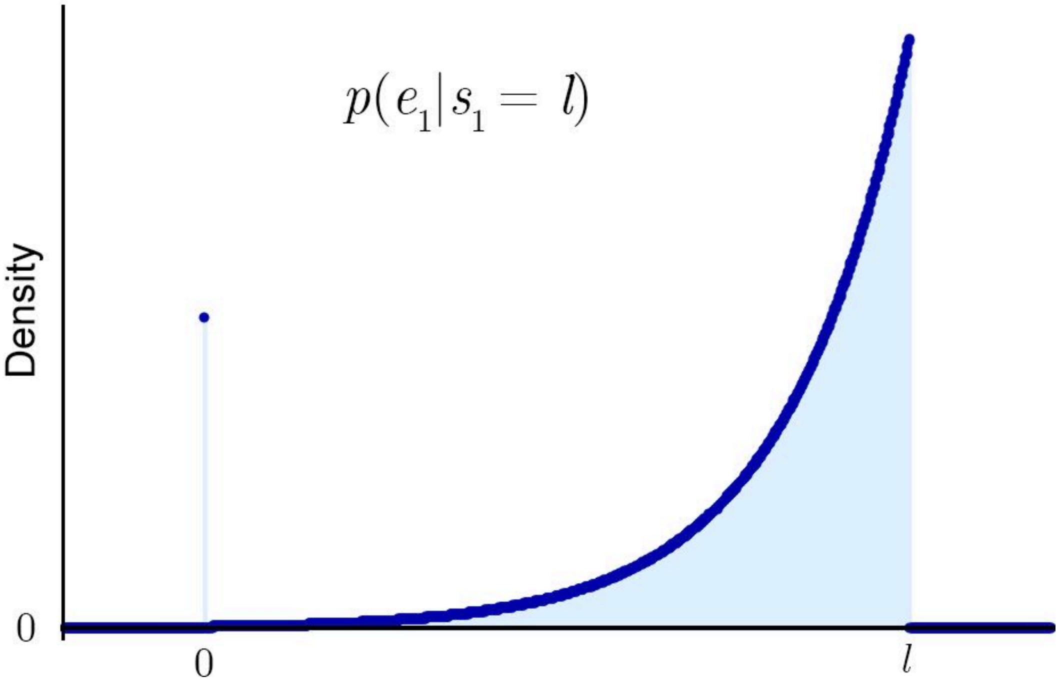

Figure S2. Related to Figure 1. Related to Derivation of models (method details). (**a**) Illustration of mRNA and first- and second-strand synthesis. (**b**) Graph of a typical stopping density for first-strand synthesis. If synthesis is based on poly-A tail priming, stopping positions decrease exponentially from the 3' end until the 5' end is reached, giving rise to a spike at the 5' end (position 0). The density equals zero outside the transcript length. Analogous the graph for $p(e_2 \mid s_2)$, mirrored horizontally and with different starting positions.

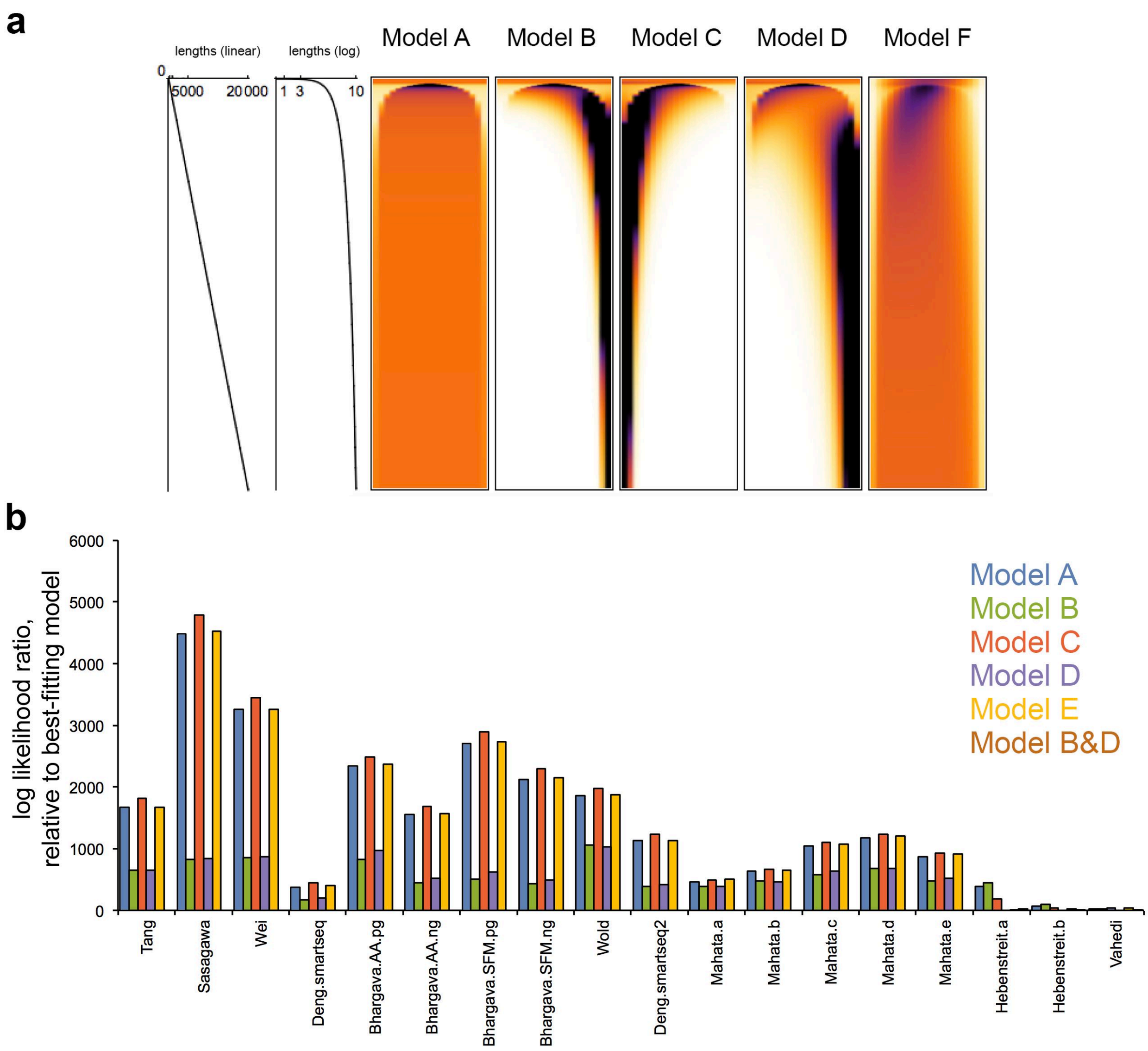

Figure S3. Related to Figure 2. (**a**) Coverage heatmaps for the different models A to E as in Figure 1C, using a transcript length distribution that linearly increases from 1 bp to 20 kb (bottom row). (**b**) Log likelihood ratios with respect to the best-fitting models for all models and relevant datasets used in this study. Best-fitting model in most cases is model B&D. The lower the value, the better the fit compared to the best model.

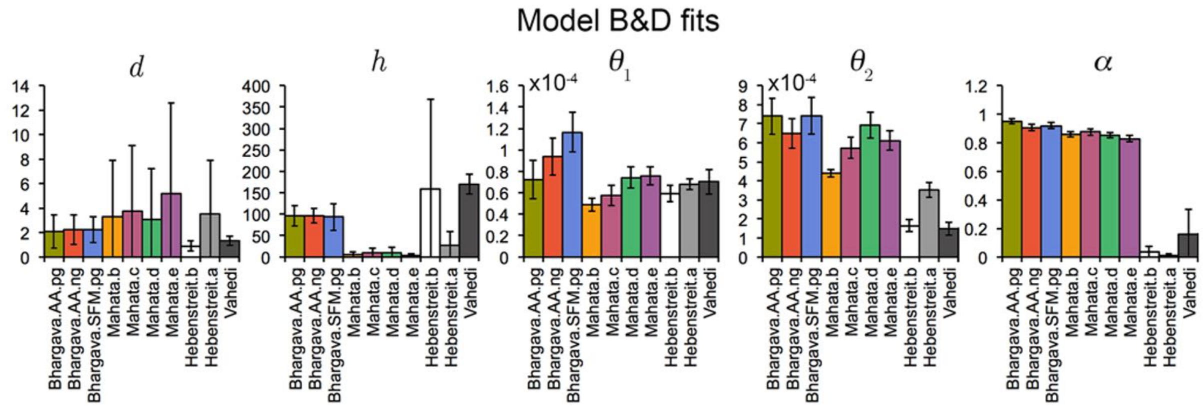

Figure S4. Related to Figure 2. MCMC parameter estimates for model B&D for datasets not shown in Figure 2C.The bar heights correspond to the medians, the error bars correspond to the median absolute deviations.

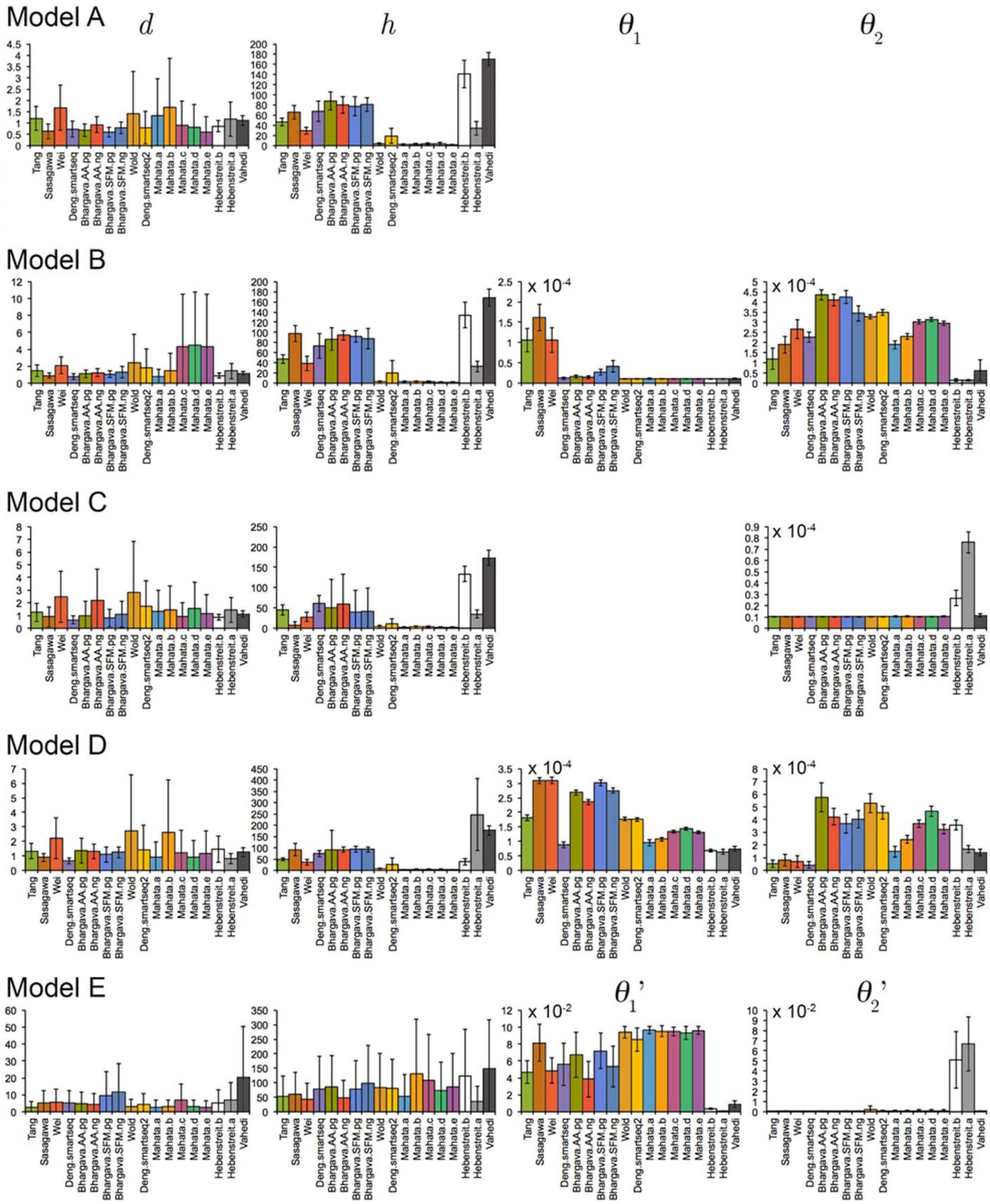

Figure S5. Related to Figure 2. MCMC parameter estimates for all relevant datasets and models except model B&D (Figure 2C;Figure S4). The bar heights correspond to the medians, the error bars correspond to the median absolute deviations.

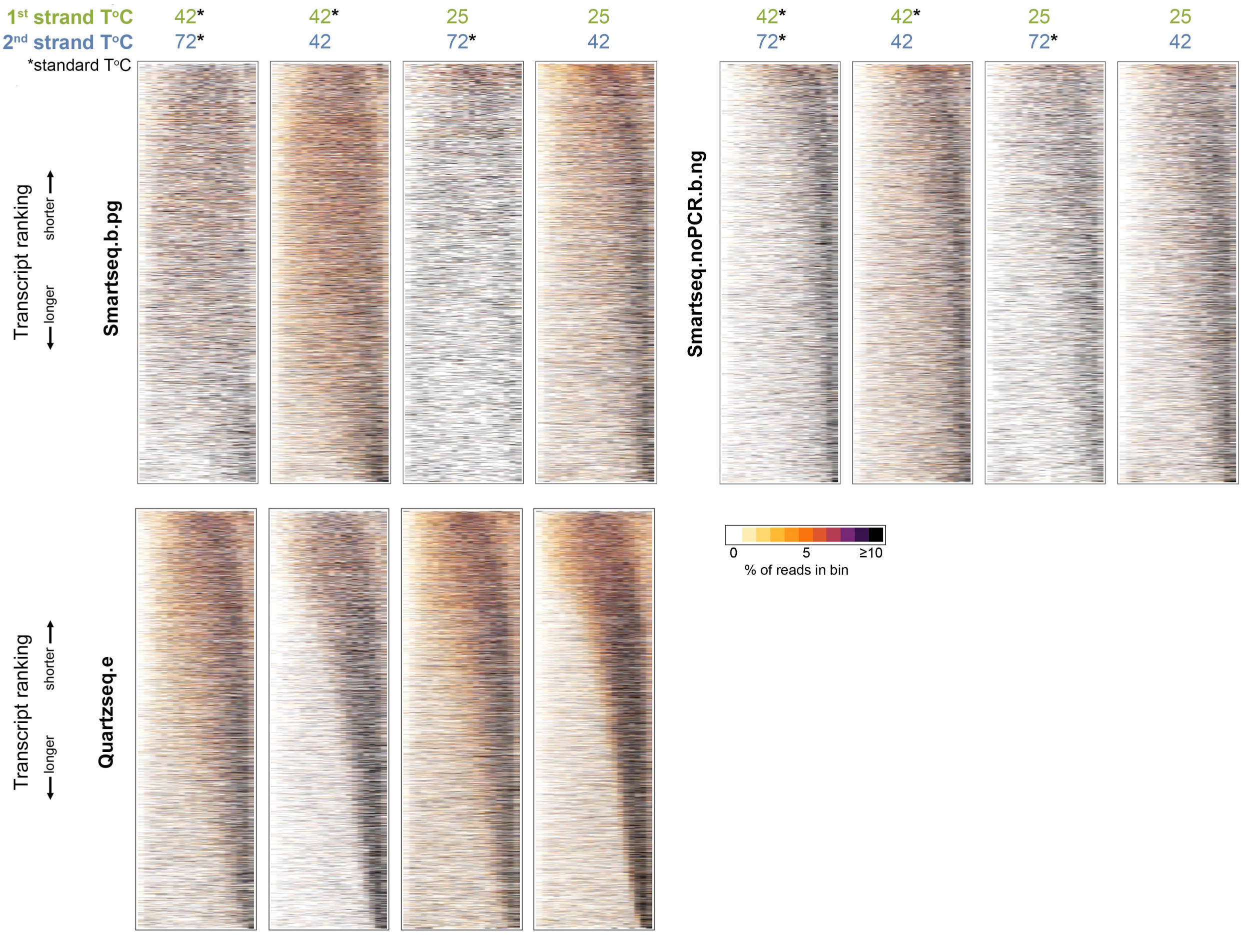

Figure S6. Related to Figure 3. Coverage heatmaps as in Figure 1C for a selection of RNA-seq samples prepared in this study as indicated. Transcripts are ordered by length. Four different combinations of incubation temperatures for first- and second-strand synthesis were used as indicated. 'Standard' temperatures are 42°C and 72°C for first- and second-strand, respectively, which were lowered to 25°C and 42°C, respectively, in the designated samples.

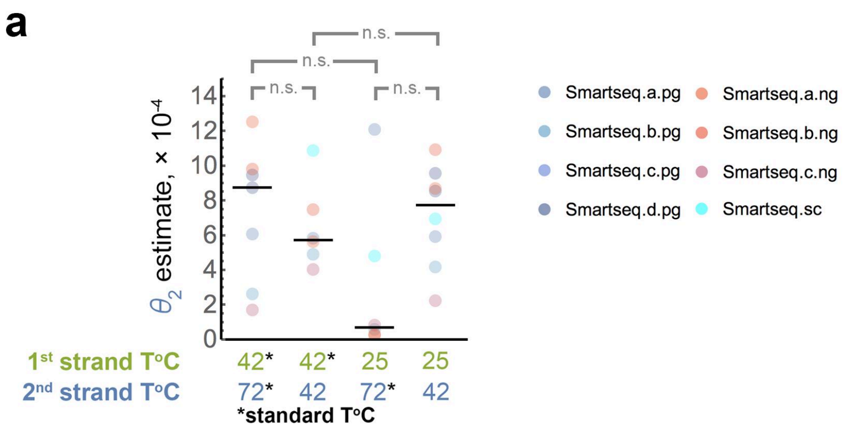

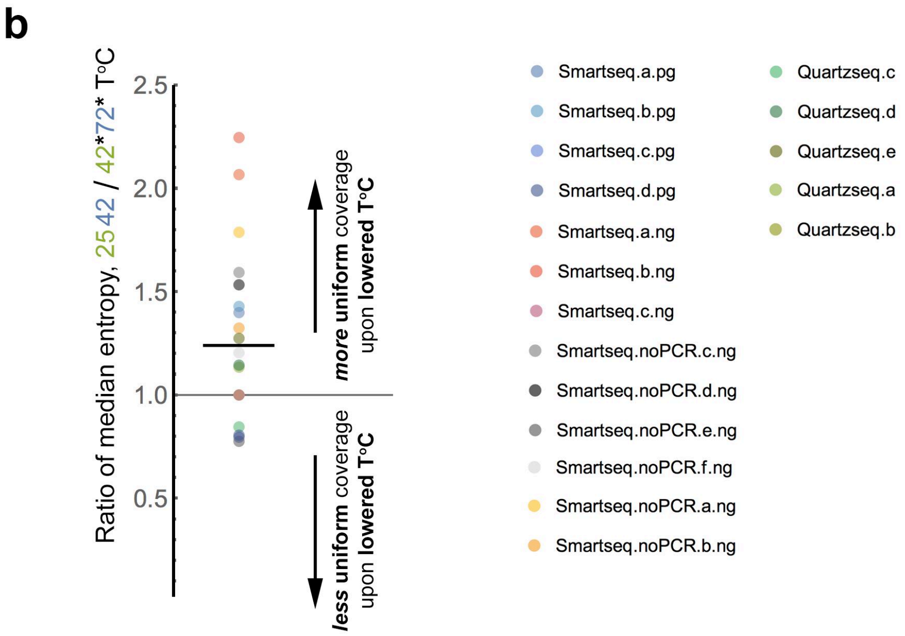

Figure S7. Related to Figure 3; Figure 4. (**a**) MCMC parameter estimates for Θ_2 for diverse SMART-seq samples (Table S1, color code on right of figure) prepared with altered reaction temperatures during first- and second-strand syntheses. 'Standard' temperatures were 42°C and 72°C for first- and second-strand, respectively, which were lowered to 25°C and 42°C, respectively, in the designated samples (black horizontal lines indicate the median; n.s., one-sided Wilcoxon signed-rank tests). (**b**) Ratios of the average coverage uniformities between lowered incubation temperatures for first- and second-strand (25°C and 42°C, respectively) and 'standard' temperatures (42°C and 72°C, respectively). Sequencing reads along transcripts were binned in the same way as for the heatmaps (e.g. Figure 1c), and statistical entropy (as a measure of uniformity) was calculated for each transcript. The statistical entropy becomes maximal for uniform distributions. The medians of the resulting distributions were determined and are shown as ratios for the RNA-seq samples as indicated.

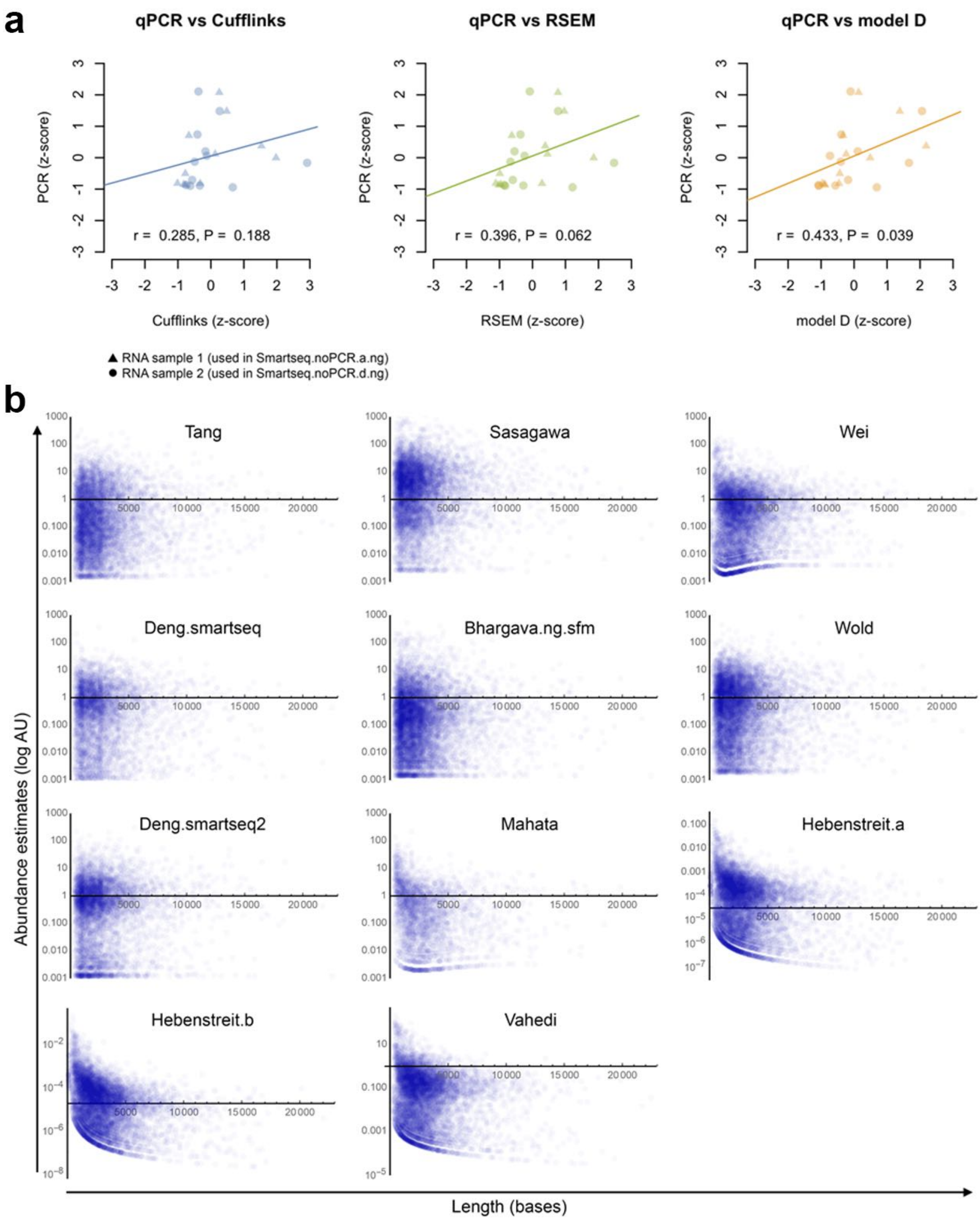

Figure S8. Related to Figure 4. (**a**) Comparison of RNA-seq abundance estimates with qPCR-based quantification. RNA-seq samples deriving from two different RNA samples (Table S1) were quantified by RSEM, Cufflinks, or our Model D as indicated for twelve different genes that cover a wide range of mRNA lengths (Table S2). In parallel, the corresponding two RNA samples (dots and triangle symbols, respectively) were subjected to qPCR for the same twelve genes. Each dot corresponds to one gene. Fitted trend lines and

Pearson's product moment correlation coefficient (*r*) and its *P*-value are shown. Standardized measures (z-scores) are used to make the approaches comparable. (**b**) Abundance estimates vs. transcript lengths based on fitting our models. Vahedi (RNA-fragmentation) was fit with model A, Hebenstreit.a/b (random priming) with model E, all others (poly-A-tagging and SMART-seq) with model B&D.

SUPPLEMENTAL TABLES

Sample name	1st strand T°C	2nd strand T°C	Protocol	Tissue	Starting Material	used in **qPCR & RNA-seq analysis**	Used in θ_1 **pertur-bation analysis**	Used in θ_2 **pertur-bation analysis**	Used in **sensitivity analysis** (spike-in probes)	Used in **length ratio & local bias analysis**	**Read numbers** $(\times 10^4)$
Quartzseq.a.r25ss42	25	42	Quartz-seq	Liver	100 ng Poly(A)+		x	x	x	x	903
Quartzseq.a.r25ss72	25	72	Quartz-seq	Liver	100 ng Poly(A)+		x	x			827
Quartzseq.a.r42ss42	42	42	Quartz-seq	Liver	100 ng Poly(A)+		x	x			501
Quartzseq.a.r42ss72	42	72	Quartz-seq	Liver	100 ng Poly(A)+		x	x	x	x	801
Quartzseq.b.r25ss42	25	42	Quartz-seq	Lymphocyte	100 ng Poly(A)+		x	x	x	x	1051
Quartzseq.b.r25ss72	25	72	Quartz-seq	Lymphocyte	100 ng Poly(A)+		x	x			1452
Quartzseq.b.r42ss42	42	42	Quartz-seq	Lymphocyte	100 ng Poly(A)+		x	x			0.44
Quartzseq.b.r42ss72	42	72	Quartz-seq	Lymphocyte	100 ng Poly(A)+		x	x	x	x	1554
Quartzseq.c.r25ss42	25	42	Quartz-seq	Lymphocyte	100 ng Poly(A)+		x	x	x	x	1691
Quartzseq.c.r25ss72	25	72	Quartz-seq	Lymphocyte	100 ng Poly(A)+		x	x			1447
Quartzseq.c.r42ss42	42	42	Quartz-seq	Lymphocyte	100 ng Poly(A)+		x	x			1922
Quartzseq.c.r42ss72	42	72	Quartz-seq	Lymphocyte	100 ng Poly(A)+		x	x	x	x	813
Quartzseq.d.r25ss42	25	42	Quartz-seq	Lymphocyte	100 ng Poly(A)+		x	x	x	x	2407
Quartzseq.d.r25ss72	25	72	Quartz-seq	Lymphocyte	100 ng Poly(A)+		x	x			2519
Quartzseq.d.r42ss42	42	42	Quartz-seq	Lymphocyte	100 ng Poly(A)+		x	x			649
Quartzseq.d.r42ss72	42	72	Quartz-seq	Lymphocyte	100 ng Poly(A)+		x	x	x	x	1753
Quartzseq.e.r25ss42	25	42	Quartz-seq	Lymphocyte	100 ng Poly(A)+		x	x	x	x	1354
Quartzseq.e.r25ss72	25	72	Quartz-seq	Lymphocyte	100 ng Poly(A)+		x	x			1255
Quartzseq.e.r42ss42	42	42	Quartz-seq	Lymphocyte	100 ng Poly(A)+		x	x			743
Quartzseq.e.r42ss72	42	72	Quartz-seq	Lymphocyte	100 ng Poly(A)+		x	x	x	x	1196
Smartseq.a.ng.r25ss42	25	42	Smart-seq	Lymphocyte	100 ng Poly(A)+		x		x	x	894
Smartseq.a.ng.r25ss72	25	72	Smart-seq	Lymphocyte	100 ng Poly(A)+		x				729
Smartseq.a.ng.r42ss42	42	42	Smart-seq	Lymphocyte	100 ng Poly(A)+		x				264
Smartseq.a.ng.r42ss72	42	72	Smart-seq	Lymphocyte	100 ng Poly(A)+		x		x	x	114
Smartseq.a.pg.r25ss42	25	42	Smart-seq 2	Liver	10 pg Total RNA		x		x	x	2297
Smartseq.a.pg.r25ss72	25	72	Smart-seq 2	Liver	10 pg Total RNA		x				928
Smartseq.a.pg.r42ss42	42	42	Smart-seq 2	Liver	10 pg Total RNA		x				3956
Smartseq.a.pg.r42ss72	42	72	Smart-seq 2	Liver	10 pg Total RNA		x		x	x	1481
Smartseq.b.ng.r25ss42	25	42	Smart-seq	Lymphocyte	100 ng Poly(A)+		x		x	x	1487
Smartseq.b.ng.r25ss72	25	72	Smart-seq	Lymphocyte	100 ng Poly(A)+		x				1190
Smartseq.b.ng.r42ss42	42	42	Smart-seq	Lymphocyte	100 ng Poly(A)+		x				1172
Smartseq.b.ng.r42ss72	42	72	Smart-seq	Lymphocyte	100 ng Poly(A)+		x		x	x	622
Smartseq.b.pg.r25ss42	25	42	Smart-seq 2	Liver	10 pg Total RNA		x		x	x	977
Smartseq.b.pg.r25ss72	25	72	Smart-seq 2	Liver	10 pg Total RNA		x				380
Smartseq.b.pg.r42ss42	42	42	Smart-seq 2	Liver	10 pg Total RNA		x				893
Smartseq.b.pg.r42ss72	42	72	Smart-seq 2	Liver	10 pg Total RNA		x		x	x	312
Smartseq.c.ng.r25ss42	25	42	Smart-seq	Lymphocyte	100 ng Poly(A)+		x		x	x	37
Smartseq.c.ng.r25ss72	25	72	Smart-seq	Lymphocyte	100 ng Poly(A)+		x				1013
Smartseq.c.ng.r42ss42	42	42	Smart-seq	Lymphocyte	100 ng Poly(A)+		x				639
Smartseq.c.ng.r42ss72	42	72	Smart-seq	Lymphocyte	100 ng Poly(A)+		x		x	x	9
Smartseq.c.pg.r25ss42	25	42	Smart-seq 2	Liver	10 pg Total RNA		x		x	x	1159
Smartseq.c.pg.r42ss72	42	72	Smart-seq 2	Liver	10 pg Total RNA		x		x	x	871
Smartseq.d.pg.r25ss42	25	42	Smart-seq 2	Liver	10 pg Total RNA		x		x	x	2730
Smartseq.d.pg.r42ss72	42	72	Smart-seq 2	Liver	10 pg Total RNA		x		x	x	1200
Smartseq.noPCR.a.ng.r25ss42	25	42	Smart-seq, no PCR	Lymphocyte	100 ng Poly(A)+	x	x	x	x	x	1530
Smartseq.noPCR.a.ng.r25ss72	25	72	Smart-seq, no PCR	Lymphocyte	100 ng Poly(A)+	x	x	x			445

Smartseq.noPCR.a.ng.r42ss42	42	42	Smart-seq, no PCR	Lymphocyte	100 ng Poly(A)+	x	x	x			836
Smartseq.noPCR.a.ng.r42ss72	42	72	Smart-seq, no PCR	Lymphocyte	100 ng Poly(A)+	x	x	x	x	x	452
Smartseq.noPCR.b.ng.r25ss42	25	42	Smart-seq, no PCR	Lymphocyte	100 ng Poly(A)+		x	x	x	x	1166
Smartseq.noPCR.b.ng.r25ss72	25	72	Smart-seq, no PCR	Lymphocyte	100 ng Poly(A)+		x	x			893
Smartseq.noPCR.b.ng.r42ss42	42	42	Smart-seq, no PCR	Lymphocyte	100 ng Poly(A)+		x	x			1767
Smartseq.noPCR.b.ng.r42ss72	42	72	Smart-seq, no PCR	Lymphocyte	100 ng Poly(A)+		x	x	x	x	1836
Smartseq.noPCR.c.ng.r25ss42	25	42	Smart-seq, no PCR	Lymphocyte	1 μg Total RNA		x	x		x	13523
Smartseq.noPCR.c.ng.r25ss72	25	72	Smart-seq, no PCR	Lymphocyte	1 μg Total RNA		x	x			3996
Smartseq.noPCR.c.ng.r42ss42	42	42	Smart-seq, no PCR	Lymphocyte	1 μg Total RNA		x	x			4689
Smartseq.noPCR.c.ng.r42ss72	42	72	Smart-seq, no PCR	Lymphocyte	1 μg Total RNA		x	x		x	10196
Smartseq.noPCR.d.ng.r25ss42	25	42	Smart-seq, no PCR	Lymphocyte	100 ng Poly(A)+	x	x	x		x	5744
Smartseq.noPCR.d.ng.r25ss72	25	72	Smart-seq, no PCR	Lymphocyte	100 ng Poly(A)+	x	x	x			2880
Smartseq.noPCR.d.ng.r42ss42	42	42	Smart-seq, no PCR	Lymphocyte	100 ng Poly(A)+	x	x	x			5635
Smartseq.noPCR.d.ng.r42ss72	42	72	Smart-seq, no PCR	Lymphocyte	100 ng Poly(A)+	x	x	x		x	2420
Smartseq.noPCR.e.ng.r25ss42	25	42	Smart-seq, no PCR	Lymphocyte	100 ng Poly(A)+		x	x		x	3598
Smartseq.noPCR.e.ng.r25ss72	25	72	Smart-seq, no PCR	Lymphocyte	100 ng Poly(A)+		x	x			3558
Smartseq.noPCR.e.ng.r42ss42	42	42	Smart-seq, no PCR	Lymphocyte	100 ng Poly(A)+		x	x			4226
Smartseq.noPCR.e.ng.r42ss72	42	72	Smart-seq, no PCR	Lymphocyte	100 ng Poly(A)+		x	x		x	2753
Smartseq.noPCR.f.ng.r25ss42	25	42	Smart-seq, no PCR	Lymphocyte	100 ng Poly(A)+		x	x		x	3424
Smartseq.noPCR.f.ng.r25ss72	25	72	Smart-seq, no PCR	Lymphocyte	100 ng Poly(A)+		x	x			4847
Smartseq.noPCR.f.ng.r42ss42	42	42	Smart-seq, no PCR	Lymphocyte	100 ng Poly(A)+		x	x			3639
Smartseq.noPCR.f.ng.r42ss72	42	72	Smart-seq, no PCR	Lymphocyte	100 ng Poly(A)+		x	x		x	7234
Smartseq.sc.r25ss42	25	42	Smart-seq 2	CD5+ lymphocyte	Single cell		x			x	224
Smartseq.sc.r25ss72	25	72	Smart-seq 2	CD5+ lymphocyte	Single cell		x				236
Smartseq.sc.r42ss42	42	42	Smart-seq 2	CD5+ lymphocyte	Single cell		x				179
Smartseq.sc.r42ss72	42	72	Smart-seq 2	CD5+ lymphocyte	Single cell		x			x	0.62

Table S1. Related to Figure 3. RNA-seq samples prepared in this study.

ID	mRNA length (bases)	fwd primer	rev primer	amplicon length (bases)	distance to 3' end of mRNA (bases)
NM_001100181.1	1913	cctcctcatcccctctcttc	gagttttggagcccatggtac	178	178
NM_001100181.1	1913	cctcctcatcccctctcttc	agttttggagcccatggtaca	177	178
NM_026389.3	1951	ggagcttctttcaggccaac	gcaagaatgaaaggacctctaac	108	131
NM_026389.3	1951	ggagcttctttcaggccaac	gtgaaggaatcctaagacctggg	72	131
NM_025989.3	1978	ttcgattgccctggaaatgc	gcaagggttgatgtattcaag	143	146
NM_025989.3	1978	ttcgattgccctggaaatgc	tgggatttgccctttcccat	83	146
NM_027949.1	1984	gcccaggacagtgagataca	ggagagatgacctttatttgtc	226	226
NM_027949.1	1984	gcccaggacagtgagataca	aggaagcacatgagagccac	178	226
NM_001081061.1	9921	cccaggcaagacatagatgc	ctacagaacattactgctttctttag	162	199
NM_001081061.1	9921	cccaggcaagacatagatgc	acatggtcacttgcatttgaataa	72	199
NM_013889.2	9977	tggattgtgttgagtagttggt	aaatcaaccatgaaaaccacc	133	151
NM_013889.2	9977	tggattgtgttgagtagttggt	acagtgggcttaccaaggat	90	151
NM_001160400.1	10040	gcttgggactcttgctttcc	gtcttctccacactttattctttg	195	197
NM_001160400.1	10040	gcttgggactcttgctttcc	tgacccatgctgacatgcac	143	197
NM_001081203.1	10070	cagggttcatttgcccacaa	caaagtgcatttgtttaattttatttaagaactttac	156	156
NM_054053.4	19327	gcattgcttagtgtttgtgca	ggattccaatacttttattgatg	171	171

NM_054053.4	19327	gcattgcttagtgtttgtgca	accatgttagccaagttcgga	134	171
NM_054053.4	19327	gcattgcttagtgtttgtgca	gattattttcattaaaccatgttagcc	149	171
NM_001033276.3	19825	ccctgtccttggcatgtttt	gtttaaaaacaaactttgaagaagcaaaatcc	114	142
NM_001033276.3	19825	ccctgtccttggcatgtttt	ccaaaacttgccctttgcct	83	142
NM_001005510.2	21718	ggtctggttgagctgtttgg	gagaaacatctgagttgacatatc	81	112
NM_010889.1	22489	accaaaccagtccttcccaa	gttgcccatgaacagtttatttc	210	210
NM_010889.1	22489	accaaaccagtccttcccaa	aaacaagggaaagggcttct	95	210

Table S2. Related to Figure 4; Related to Key Resources Table. Genes and corresponding primers used in qPCR.
